# Enhanced interactions within microenvironment accelerates dismal prognosis in HBV-related HCC after TACE

**DOI:** 10.1097/HC9.0000000000000548

**Published:** 2024-10-03

**Authors:** Libo Wang, Jiahui Cao, Zaoqu Liu, Shitao Wu, Yin Liu, Ruopeng Liang, Rongtao Zhu, Weijie Wang, Jian Li, Yuling Sun

**Affiliations:** 1 1 Department of Hepatobiliary and Pancreatic Surgery, The First Affiliated Hospital of Zhengzhou University, Zhengzhou, Henan Province, China; 2 2 Department of Pancreatic Cancer, Tianjin Medical University Cancer Institute and Hospital, National Clinical Research Center for Cancer, Key Laboratory of Cancer Prevention and Therapy, Tianjin, China; 3 3 Institute of Basic Medical Sciences, Chinese Academy of Medical Sciences and Peking Union Medical College, Beijing, China; 4 4 Institute of Hepatobiliary and Pancreatic Diseases, Zhengzhou University, Zhengzhou, Henan Province, China; 5 5 Zhengzhou Basic and Clinical Key Laboratory of Hepatopancreatobiliary Diseases, The First Affiliated Hospital of Zhengzhou University, Zhengzhou, Henan Province, China

## Abstract

**Background:**

Transarterial chemoembolization (TACE) is the first-line treatment for patients with advanced HCC, but there are limited studies on the microenvironment alterations caused by TACE.

**Methods:**

Six fresh HBV-related HCC specimens with or without TACE intervention were used to perform single-cell RNA sequencing. The 757 bulk samples from 3 large-scale multicenter cohorts were applied for comprehensive analysis. The biological functions of the biomarkers were further validated by phenotypic experiments.

**Results:**

Using single-cell RNA sequencing analysis, we delineated the global cell atlas of post-TACE and demonstrated elevated tumor heterogeneity and an enhanced proinflammatory microenvironment induced by TACE. Cell-cell communication analysis revealed that markedly elevated interactions between NABP1+ malignant hepatocytes, neutrophils, and CD8+ T cells after TACE might accelerate the shift from CD8+ effector memory T cells to CD8+ effector T cells. This result was substantiated by the developmental trajectory between the 2 and dramatically decreased resident scores along the pseudotemporal trajectory. Integrating bulk data, we further found that the increased estimated proportion of NABP1+ malignant hepatocytes was related to poor TACE response and dismal prognosis, and its biomarker role could be replaced by NABP1. In vitro, multiple biological experiments consistently verified that NABP1 knockdown significantly inhibited the proliferation and migration of HCC cells.

**Conclusions:**

Based on our depicted global map of post-TACE, we confirmed that the enhanced interactions within the microenvironment after TACE may be the culprits for postoperative progression. NABP1 may become an attractive tool for the early identification of patients sensitive to first-line TACE in clinical practice.

## INTRODUCTION

As the sixth most prevalent tumor, primary liver cancer has the third highest mortality rate, and HCC is the most dominant histological type.^1,2^ Attributed to the high infection rate of HBV, ~85% of HCC cases in China are HBV-related HCC.^3^ Over the past decade, immunotherapies, represented by immune checkpoint inhibitors, have largely revolutionized the treatment paradigm for solid tumors, yet only 25% of patients with HCC experience durable responses.^4^ With recent technological advances, for some patients with intermediate-stage to advanced HCC, conversion therapy can lead to tumor shrinkage or downstaging followed by radical resection to achieve long-term survival.^5^ Currently, the major methods of conversion therapy are locoregional treatment, such as transarterial chemoembolization (TACE), systemic antitumor therapy, and other strategies for residual liver deficiency.^5^

In Western countries, TACE is recommended as the first-line therapy for intermediate-stage (Barcelona Clinic Liver Cancer) HCC and as a neoadjuvant therapy for patients whose tumors can be resected after downstaging.^6^ In China, TACE is gradually becoming the preferred regimen for advanced HCC.^5^ A recent meta-analysis demonstrated that preoperative TACE combined with hepatectomy significantly extended relapse-free survival and resulted in less intraoperative blood loss than surgery alone.^7^ However, the current understanding of TACE-driven changes in the tumor microenvironment (TME) is very limited, and only a few studies have focused on developing biomarkers available for diagnosis and follow-up through exome or whole-genome sequencing of plasma and needle biopsy samples.^6,8–11^ In one of the few studies that conducted bulk transcriptome sequencing on HCC tissues after TACE, Zhong et al^12^ proved that S100A9 was a critical driver of progression after TACE. Moreover, Pinato et al^13^ also confirmed that preoperative TACE resulted in a reduction in both immune-exhausted effector cells and regulatory T cells within the TME and the upregulation of proinflammatory pathways, which provided a rationale for immunotherapy together with TACE.

With the rapid improvement and increasing accessibility of single-cell RNA sequencing (scRNA-seq), the TME of HCC, HBV-related HCC, early-relapse HCC, and immunotherapy-sensitive/insensitive HCC has been greatly elucidated.^14–19^ Recently, Tan et al^20^ revealed that TREM2+ macrophages could inhibit the infiltration of CD8+ T cells and promote the progression of HCC by performing scRNA-seq on CD45+ immune cells after TACE in 5 patients with HCC. However, there are still few studies regarding the heterogeneous changes in HCC tumor cell subsets, the interactions between tumor cells and the microenvironment, and the evident differences in TACE sensitivity among patients with HCC after TACE intervention. Residual disease and relapse remain major bottlenecks limiting the efficacy of TACE.^21^

In this study, we aimed to perform scRNA-seq analysis on patients with HCC with and without TACE to elucidate the changes in the microenvironment after TACE and the crosstalk between different components. In addition, we further confirmed through in vitro experiments that NABP1, with dramatically increased expression after TACE, promoted HCC proliferation and migration, and was correlated with a worse TACE response and dismal outcome.

## METHODS

### Patient samples

A total of 6 samples from 3 newly diagnosed patients with HCC who underwent surgery and 3 patients with HCC who underwent TACE for 1–2 months followed by surgery between March 2022 and October 2022 at the First Affiliated Hospital of Zhengzhou University were subjected to scRNA-seq. All patients tested positive for HBsAg preoperatively, had not received targeted therapy, immunotherapy, and other treatments except TACE before operation, and were pathologically confirmed HCC. The study was approved by the Human Ethics Committee of the hospital (2022-KY-1519), and written informed consent was obtained from each patient. The detailed clinical characteristics of the 6 patients with HCC are shown in Supplemental Table S1, http://links.lww.com/HC9/B48.

### Single-cell RNA sequencing

Following the standard pipeline of 10X Genomics, we selected suspensions with cell viability >90%, clumping rate <5%, and debris-free for subsequent sequencing. Next, we performed scRNA-seq through a 4-step pipeline. (1) For reverse transcription, Gel bead-in-EMulsion (GEM) was prepared by adding qualified single-cell suspension, gel bead with 10X barcode, and oil to different chambers of the Chromium Next Gem Chip G Single Cell Kit. Following this, GEM was loaded on the PCR instrument for reverse transcription. Purified cDNA strands were amplified by PCR and then tested for concentration and quality control (QC) using a nucleic acid quantifier. (2) For library construction, after the qualified cDNA was enzymatically cut and segmented, the most suitable fragments were purified and selected by magnetic beads. Then, the cDNA library was constructed by end repair, 3′ end polyadenylation, adapter ligation, library amplification, and other steps. (3) For sequencing, according to the guidelines provided by Illumina Sequencer, cluster generation and first-way sequencing primer hybridization steps were performed, followed by loading the flowcell containing the clusters onto the sequencer for paired-end sequencing. (4) For the CellRanger count, the raw fastq files were converted to a feature-barcode expression matrix using CellRanger (v5.0.0) software and loaded into R software for subsequent analysis.

### Determination of cell type and clustering

Referring to previous studies,^14,18–20^ we conducted a 4-step pipeline to analyze the scRNA-seq data. (1) For QC and standardization, after the initial filtering of the expression matrix of individual samples, 6 samples from the TACE and HCC groups were merged. Next, we performed secondary filtering based on the following criteria: (a) the number of genes per cell, nFeature_RNA, was >100 but <5000; (b) the total number of unique molecular identifiers per cell was >500 but <25,000; and (c) the percentage of mitochondrial genes was below 25%. (2) For integration and dimensionality reduction, after removing batch effects for 6 samples using the “anchor” strategy developed by the Seurat team,^22^ which is based on canonical correlation analysis and mutual nearest neighbors algorithms, we performed dimensionality reduction analysis on the integrated data using the principal component analysis algorithm. (3) For cell clustering and visualization, we used the FindNeighbors and FindClusters functions to perform clustering analysis on the normalized data after integration, and visualized the clustering results by the uniform manifold approximation and projection algorithm. (4) For cell annotation, the *SingleR* package^23^ was used to automatically annotate cell clusters, and we also manually corrected the above results by referring to the cell-type markers reported in the literature.^16–20^

### Pseudotime analysis

To better delineate the cellular differentiation states between the distinctly altered T-cell subsets caused qby TACE, we applied the *Monocle 3* package for trajectory inference and pseudotime analysis.^24^ In addition, the CytoTRACE computational framework can robustly predict the differentiation states, and the developmental potential of scRNA-seq data was used to assist in inferring the starting point of pseudotemporal cells.^25^

### Cell-cell communication

CellChat, an intercellular communication analysis tool for ligands, receptors, and their cofactors, was employed to quantitatively infer and analyze the cell-to-cell interaction networks between malignant hepatocytes and notably altered myeloid cell subsets, as well as myeloid cells and different state T-cell subsets.^26^

### Pathway enrichment analysis

To gain insight into the underlying biological behavior of malignant hepatocytes, based on the Hallmark, Gene Ontology and Kyoto Encyclopedia of Genes and Genomes (KEGG) gene sets, we conducted gene set variation analysis and gene set enrichment analysis pathway enrichment analysis at the single-cell level.

### Bulk data acquisition and processing

#### Bulk RNA-seq data

The FPKM expression data and survival information for The Cancer Genome Atlas (TCGA)-LIHC (n = 368) were obtained from the UCSC Xena (http://xena.ucsc.edu/) website.

#### Bulk microarray data

The robust multi-array average-normalized microarray data, survival, and TACE response information for GSE14520 (n = 242) and GSE104580 (n = 147) were downloaded directly from the Gene Expression Omnibus website (https://www.ncbi.nlm.nih.gov/geo/) for subsequent analysis.

### Cell culture and transfection

Human HCC (HepG2) cells were maintained in DMEM (Solarbio) containing 10% fetal bovine serum at 37°C and 5% CO_2_. The 3 si-NABP1 sequences purchased from RiboBio are shown in Supplemental Table S2, http://links.lww.com/HC9/B48. Using Lipofectamine 3000 (Invitrogen) as a carrier, we transfected si-NC and si-NABP1 into HCC cells, which were harvested 48 hours later and verified for transfection efficiency.

### qRT-PCR and western blotting assay

The primers for quantitative real-time PCR of β‐actin and NABP1 are shown in Supplemental Table S3, http://links.lww.com/HC9/B48. Using the primary antibodies anti‐NABP1 (rabbit, 1:1000, Proteintech) and anti‐β‐actin (rabbit, 1:5000, Proteintech), we examined the expression of NABP1 and β‐actin protein through western blotting assay.

### Cell proliferation and migration assay

Cell counting kit-8 (CCK-8) and colony‐forming assays were performed to evaluate the relationship between NABP1 and cell viability. For the CCK-8 assay, cells were seeded on 96‐well plates (5 × 10^3^ per well), and the OD value at 450 nm was measured using a CCK-8 kit (TargetMol) at 0, 24, 48, 72, and 96 hours after transfection to estimate the cell proliferation index. In addition, we inoculated HepG2 cells (logarithmic growth phase, 1000 cells/well) on 6-well plates and cultured them for 2 weeks. The clone number was counted after fixing and staining cells.

Next, we performed scratch-wound healing and Transwell assays. After starving for 24 hours in serum-free medium, a 200 μL pipette tip was used to evenly scratch and wash off cell debris. The culture was continued for 48 hours with serum-free medium, and images were taken at 0 and 48 hours. For the Transwell assay, we added DMEM with 20% fetal bovine serum to the lower chamber and placed 1 × 10^5^ transfected HCC cells into the upper chamber after resuspension in 100 μL serum-free medium. After incubation for 24 hours, the upper chamber was washed twice, and residual cells were gently wiped with wet swabs. The cells were then fixed with 4% paraformaldehyde for 20 minutes and stained with 1% crystal violet for 15 minutes before photographing and counting for analysis.

### Statistical analysis

All data processing, statistical analyses, and visualizations were conducted in R 4.3.1. Student *t*-test and the Wilcoxon rank sum test were used to compare continuous variables. The receiver operating characteristic curve and the area under the receiver operating characteristic curve (AUC) were used to evaluate the predictive performance. All 2-sided *p* values <0.05 were regarded as statistically significant.

## RESULTS

### Landscape of the altered microenvironment in HCC after TACE

**FIGURE 1:**
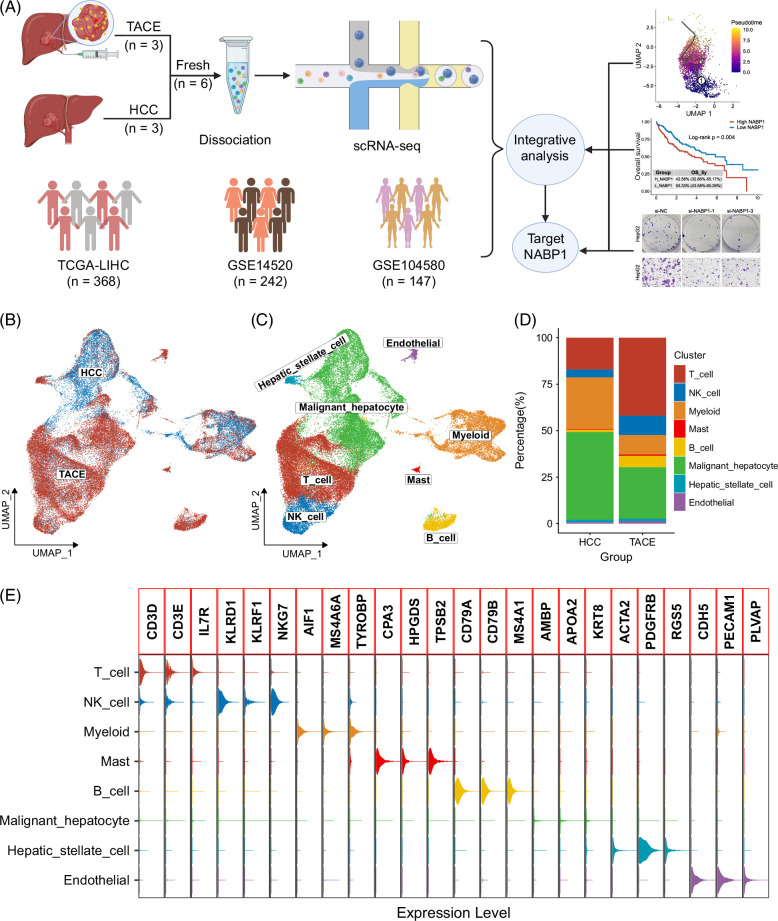
Single-cell RNA sequencing atlas of HCC with and without TACE. (A) The workflow of our study. (B) The UMAP visualization for qualified cells in the HCC and TACE groups. (C) The UMAP distribution for 8 major cell types. (D) The percentage of these 8 major cell types in the 2 groups. (E) The signature markers of these 8 major cell types. Abbreviations: TACE, transarterial chemoembolization; UMAP, uniform manifold approximation and projection.

The overall design of this study is shown in Figure 1A. After filtering and QC, we obtained 51,057 high-quality cells. In individual cells, the total number of unique molecular identifiers was strongly positively correlated with the detected genes (Supplemental Figure S1A, http://links.lww.com/HC9/B48). The dimension reduction and clustering analysis of the post-QC data showed distinct batch effects between the 2 groups and among 6 samples (Supplemental Figures S1B, C, http://links.lww.com/HC9/B48). After performing canonical correlation analysis and mutual nearest neighbors, the 24 clusters resulting from dimensionality reduction clustering were integrated well (Figure 1B, Supplemental Figures S1D, E, http://links.lww.com/HC9/B48).

Next, referring to the markers provided by previous studies,^16–20^ we annotated the 24 clusters into 8 cell types (Figure 1C). Among them, malignant hepatocytes were identified by AMBP, APOA2, and KRT8, whereas nonimmune stromal cells including HSCs were identified using ACTA2, PDGFRB, and RGS5, and endothelial cells were defined as CDH5, PECAM1, and PLVAP-expressing cells. On the other hand, infiltrating immune cells including T cells (CD3D/E and IL7R), natural killer (NK) cells (KLRD1, KLRF1, and NKG7), myeloid cells (AIF1, MS4A6A, and TYROBP), mast cells (CPA3, HPGDS, and TPSB2), and B cells (CD79A, CD79B, and MS4A1) were also clearly annotated (Figure 1E). Considering that the inferCNV algorithm has been widely used in scRNA-seq data to infer whether there are large-scale chromosomal copy number variations,^27^ the 7 malignant hepatocyte clusters possessed higher inferred copy number variations than the 5 myeloid cell clusters (Supplemental Figure S1F, http://links.lww.com/HC9/B48). Furthermore, compared with the absolute predominance of T cells, myeloid cells, and malignant hepatocytes in the HCC group, we found that the T cells, NK cells, and B cells were increased in the TACE group, while malignant hepatocytes and myeloid cells were correspondingly decreased, indicating that TACE treatment resulted in increased heterogeneity in HCC (Figure 1D, Supplemental Figures S2A–C, http://links.lww.com/HC9/B48). Moreover, the results obtained from the 2 groups also exhibited good agreement in each of the 3 samples (Supplemental Figures S2D, E, http://links.lww.com/HC9/B48). In terms of cell subsets, effector cells, such as T cells and NK cells, and B cells that can produce inflammatory mediators were markedly increased in patients with HCC after TACE (Supplemental Figures S2F, G, http://links.lww.com/HC9/B48).

### Functional diversity and phenotypic transfer of T cells induced by TACE

**FIGURE 2:**
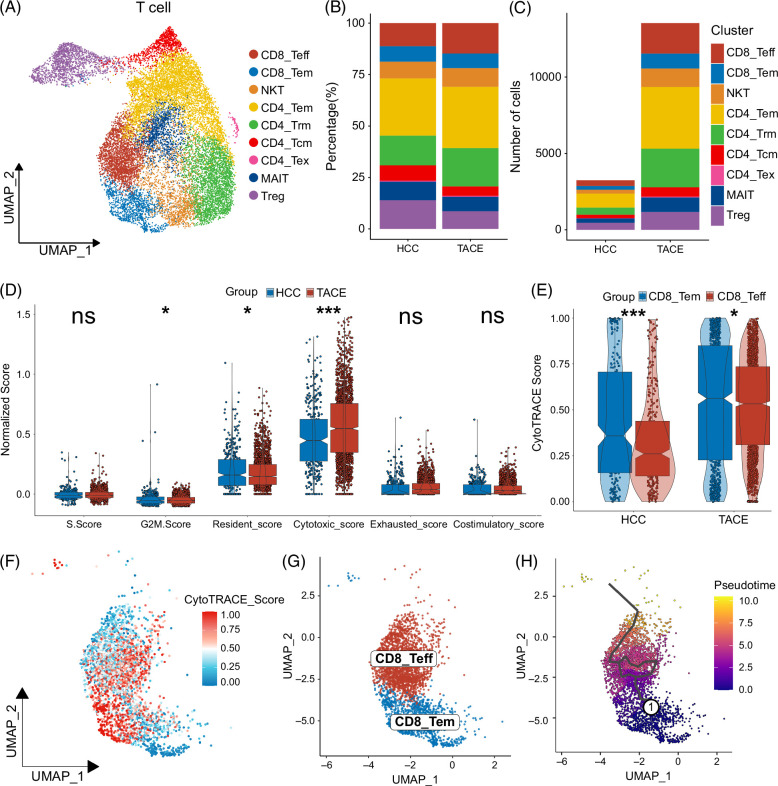
Identification and characteristics of T-cell subsets in the HCC microenvironment after TACE. (A) The UMAP distribution for 9 heterogeneous T-cell subsets. (B) The percentage of these 9 T-cell subsets in the 2 groups. (C) The numbers of these 9 T-cell subsets in the 2 groups. (D) Comparison of the S.Score, G2M.Score, and resident, cytotoxic, exhausted, and costimulatory scores of CD8+ effector T (CD8_Teff) cells in the HCC and TACE groups. (E) Comparison of the CytoTRACE scores between the CD8+ effector memory T (CD8_Tem) and CD8_Teff cells in the 2 groups. (F) The UMAP plot of CytoTRACE scores in CD8_Tem and CD8_Teff cell subsets, with higher CytoTRACE scores indicative of a more primitive differentiated state. (G) The UMAP distribution for CD8_Tem and CD8_Teff cell subsets. (H) The pseudotime trajectory for CD8_Tem and CD8_Teff cells in the 2 groups based on Monocle 3. *p < 0.05, ***p < 0.001. Abbreviations: TACE, transarterial chemoembolization; UMAP, uniform manifold approximation and projection.

Using the signatures provided by a previous study,^18^ we evaluated the cell cycle phases and functional status of 16,763 T cells. Consistent with the relatively activated TME, T cells in the TACE group possessed significantly higher S.Score, G2M.Score, and resident, cytotoxic, and costimulatory scores; however, there was no significant difference in exhausted scores (Supplemental Figures S3B–G, http://links.lww.com/HC9/B48). Then, we reclustered T cells into 17 clusters and annotated them into 9 T-cell subsets (Figure 2A, Supplemental Figure S3A, http://links.lww.com/HC9/B48).^16–20^ Among them, we detected 2 CD8+ T-cell subsets characterized by high expression of CD8A and CD8B: CD8+ effector T (CD8_Teff) cells showed increased expression of cytotoxic genes such as GZMH and GZMK, while CD8+ effector memory T (CD8_Tem) cells exhibited high expression of cytokines CCL4L2 and XCL1, and effector memory-related coactivated CD27 in addition to the above cytotoxic markers. The CD4+ T cells were defined into 4 subsets, including CD4+ effector memory T cells (CD4_Tem, GPR183, CD40LG, and IL7R), tissue-resident memory CD4+ T cells (CD4_Trm, MYADM, NR4A1, and CD69), central memory CD4+ T cells (CD4_Tcm, SELL, LEF1, and CCR7), and exhausted CD4+ T cells (CD4_Tex, TOX2, TNFRSF4, and TNFSF13B). In addition, NK T cells characterized by high expression of cytotoxic and early activated markers such as NKG7, KLRD1, and IFNG, mucosal-associated invariant T cells with specific expression of SLC4A10, KLRB1, and KLRG1, and regulatory T cells exhibiting high expression of exhausted markers such as FOXP3, TIGIT, and CTLA4, were also clearly annotated (Supplemental Figure S3H, http://links.lww.com/HC9/B48). In general, 9 T-cell subsets were evenly distributed in 6 samples, mainly as the proportion was essentially unchanged while the absolute number increased substantially (Figures 2B, C, Supplemental Figures S4A–E, http://links.lww.com/HC9/B48). Notably, among several effector cells, CD8_Teff cells had the largest changes and elevated numbers in the 2 groups and 6 samples (Supplemental Figures S4D, E, http://links.lww.com/HC9/B48).

Given the critical role of CD8+ T cells in antitumor immunity and immunotherapy, we further explored the functional heterogeneity of CD8_Teff and CD8_Tem cells. As expected, in the context of no significant difference in the S.Score and G2M.Score, the CD8_Tem cells in the TACE group had distinctly higher functional status scores (Supplemental Figure S4F, http://links.lww.com/HC9/B48). However, the G2M.Score and resident score were markedly lower in the CD8_Teff cells of patients after TACE, whereas the cytotoxic score was significantly higher (Figure 2D). Furthermore, trajectory inference confirmed that CD8_Tem cells showed a more primitive differentiation state and a pseudotemporal trajectory of development toward CD8_Teff cells, suggesting that TACE may promote the shift of CD8_Tem cells into CD8_Teff cells within the TME (Figures 2E–H). The outstanding decrease in the resident score along the pseudotemporal trajectory also confirmed the evolution of CD8+ T cells from memory to cytotoxicity (Supplemental Figure S4G, http://links.lww.com/HC9/B48).

### Different frequencies of myeloid subsets in the 2 groups

**FIGURE 3:**
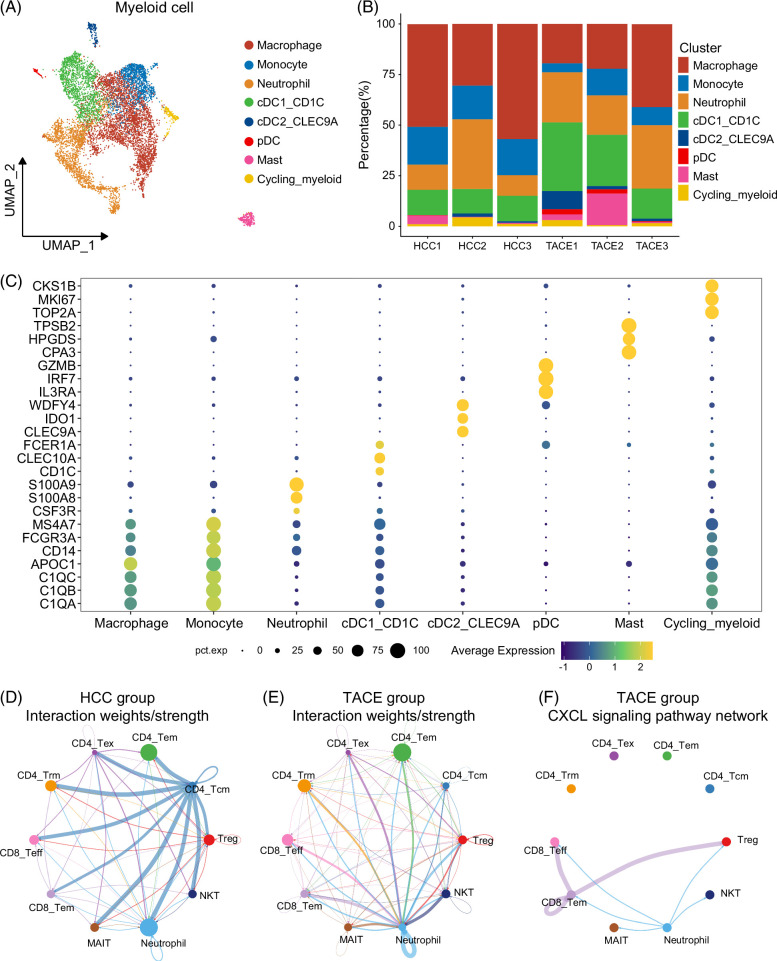
Identification and characteristics of distinct myeloid cells in the HCC microenvironment after TACE. (A) The UMAP distribution for 8 distinct myeloid cell subsets. (B) The percentage of these 8 myeloid cell subsets among the 6 samples. (C) The signature markers of these 8 myeloid cell subsets. (D, E) Interaction weights of neutrophils and 9 heterogeneous T-cell subsets in the HCC (D) and TACE (E) groups. (F) The CXCL signaling pathway network of neutrophils and 9 heterogeneous T-cell subsets in the TACE group. Abbreviations: TACE, transarterial chemoembolization; UMAP, uniform manifold approximation and projection.

In terms of myeloid cells, in contrast to T cells, the TACE group had significantly lower S.Score and G2M.Score (Supplemental Figures S5A, B, http://links.lww.com/HC9/B48). To further investigate this difference, 8483 myeloid cells were reclustered into 15 clusters, which were evenly distributed in the 2 groups (Supplemental Figures S5C, D, http://links.lww.com/HC9/B48). Using signature markers,^8,14–20^ we annotated them into 8 distinct subsets, including macrophages (C1QA/B/C and APOC1), monocytes (CD14, FCGR3A, and MS4A7), neutrophils (CSF3R, S100A8, and S100A9), 3 subtypes of dendritic cells (DCs): cDC1_CD1C (CD1C, CLEC10A, and FCER1A), cDC2_CLEC9A (CLEC9A, IDO1, and WDFY4), plasma DC (IL3RA, IRF7, and GZMB), mast cells (CPA3, HPGDS, and TPSB2), and cycling myeloid cells (TOP2A, MKI67, and CKS1B) (Figures 3A, C). In contrast to the absolute preponderance of macrophages in the HCC group, the heterogeneity of myeloid cells in the TACE group increased, as shown by a substantial increase in the proportion of several subsets, particularly neutrophils, cDC1_CD1C cells, and mast cells (Figure 3B, Supplemental Figures S5E–G, http://links.lww.com/HC9/B48).

In addition, the cell-cell communication showed that interactions between neutrophils and T-cell subsets, except for CD4_Tcm, were dramatically increased in the TACE group (Figures 3D, E). Among them, CXCL signaling pathways, such as CXCL16-CXCR6, a chemokine family that plays a crucial role in HCC carcinogenesis, progression, and metastasis,^28–31^ were markedly increased in the TACE group, especially in neutrophils and effector cells such as CD8_Tem, CD8_Teff, and NK T cells (Figure 3F, Supplemental Figures S6A–C, http://links.lww.com/HC9/B48). In detail, interactions between neutrophils and CD8_Tem and CD8_Teff cells were also increased to varying degrees (Supplemental Figures S6D–I, http://links.lww.com/HC9/B48 and Supplemental Figures S7A–D, http://links.lww.com/HC9/B48). In addition, cell-cell communication between neutrophils and other myeloid subsets revealed that interactions between neutrophils and plasma DCs and cDC2_CLEC9A cells were significantly enhanced in the TACE group, while interactions between neutrophils and cycling myeloid cells and macrophages were attenuated or even absent (Supplemental Figures S8A, B, http://links.lww.com/HC9/B48). Specifically, the interactions between neutrophils and macrophages involving IGF2-IGF2R signaling that were present in the HCC group were abolished in the TACE group, whereas there were interactions between neutrophils and monocytes and DCs through signaling pathways such as TNF and CCL (Supplemental Figures S8C–E, http://links.lww.com/HC9/B48).

### Readaptation and selection of malignant hepatocytes caused by TACE

**FIGURE 4:**
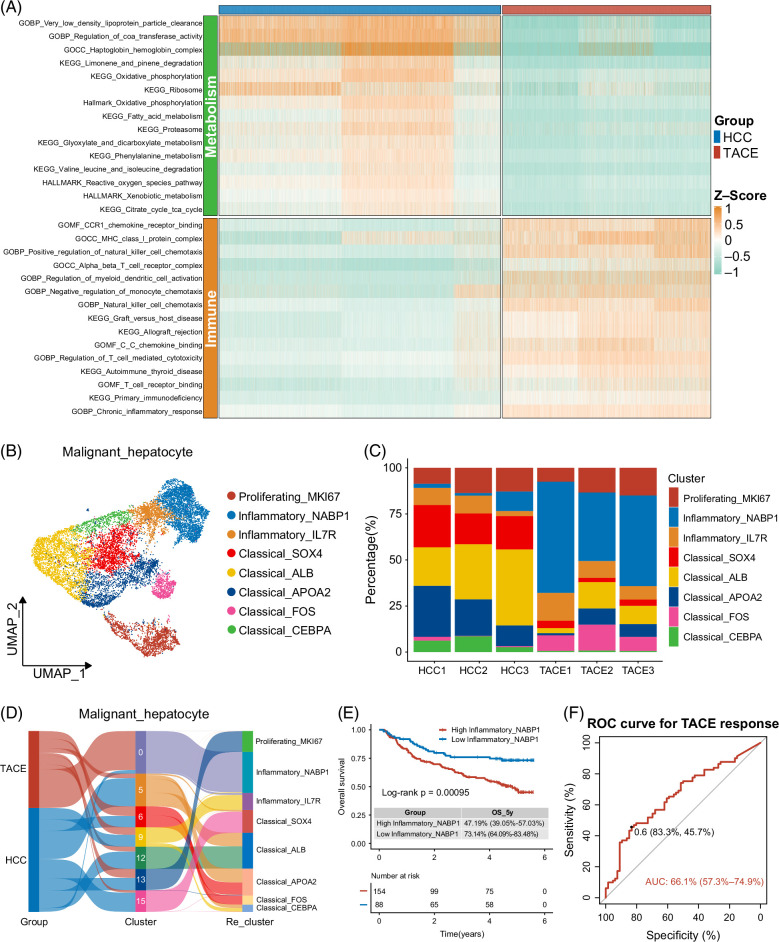
Transcriptome heterogeneity of malignant hepatocyte subsets. (A) The top 15 pathways enriched by GSVA in malignant hepatocytes of HCC and TACE groups. (B) The UMAP distribution for 8 heterogeneous malignant hepatocyte subsets. (C) The percentage of these 8 malignant hepatocyte subsets in the 6 samples. (D) The Sankey plot of 13,172 malignant hepatocytes showed their correspondence among the 2 groups, the 7 malignant hepatocyte clusters (initial clustering for all cells), and the 8 malignant hepatocyte subsets (reclustering on all malignant hepatocytes). (E) Kaplan-Meier survival curve for OS between groups with high and low estimated proportion of NABP1+ malignant hepatocytes in the GSE14520 cohort. (F) The ROC curve for TACE responsiveness predicted by the estimated proportion of NABP1+ malignant hepatocytes in the GSE104580 cohort. Abbreviations: GSVA, gene set variation analysis; OS, overall survival; ROC, receiver operating characteristic; TACE, transarterial chemoembolization; UMAP, uniform manifold approximation and projection.

The results of gene set variation analysis revealed that the HCC group was primarily enriched in metabolic pathways, such as oxidative phosphorylation and fatty acid metabolism, while the TACE group was characterized by immune-related pathways, such as MHC class-I protein complex, NK cell chemotaxis, and C-C chemokine binding (Figure 4A). In parallel, the gene set enrichment analysis also verified these functional characteristics of malignant hepatocytes in the corresponding groups (Supplemental Figures S8F, G, http://links.lww.com/HC9/B48). Subsequently, referring to the study by Liu et al,^19^ we reclustered 13,172 malignant hepatocytes into 12 clusters and annotated them into 8 subsets, including proliferating malignant hepatocytes (HMGB2, MKI67, and TOP2A), 2 subtypes of inflammatory malignant hepatocytes with PTPRC expression (NABP1/IL7R+ malignant hepatocytes), and 5 subtypes of classical malignant hepatocytes with ALB and APOA2 expression (SOX4/ALB/APOA2/FOS/CEBPA+ malignant hepatocytes) (Figure 4B, Supplemental Figures S9A, B, http://links.lww.com/HC9/B48). Moreover, all cell-based clusters in Supplemental Figure S1D, http://links.lww.com/HC9/B48, were in agreement with the 3 main subsets we reclustered. Cluster 13 corresponded to proliferating hepatocytes, clusters 0 and 9 were concentrated in inflammatory hepatocytes, and clusters 5/6/12/15 constituted classical malignant hepatocytes (Figure 4D). Compared with HCC group, the proportion of proliferating malignant hepatocytes was basically unchanged in the TACE group, the inflammatory malignant hepatocytes were significantly increased, while the classical malignant hepatocytes were significantly reduced (Figure 4C, Supplemental Figures S9C, D, http://links.lww.com/HC9/B48). In particular, only the inflammatory NABP1+ and classical FOS+ malignant hepatocytes (accounting for a very small proportion) increased dramatically when the vast majority of cells decreased in the TACE group (Supplemental Figure S9E, http://links.lww.com/HC9/B48).

### Inflammatory NABP1+ malignant hepatocytes were associated with poor TACE responsiveness and dismal prognosis

Limited to the small sample size, the relevance of scRNA-seq data to prognosis cannot be directly evaluated, and advances in bioinformatics have made it possible. Among them, Bisque is a deconvolution algorithm, which provides insights into tumor heterogeneity by comparing the bulk RNA-seq data with reference profiles of each cell subset obtained from scRNA-seq data and deconvolving them using non-negative least squares regression method to obtain the contribution ratio of each cell-type in bulk RNA-seq data.^32^ Using Bisque, we confirmed that patients with HCC with a higher estimated proportion of NABP1+ malignant hepatocytes exhibited significantly shorter OS and relapse-free survival in the GSE14520 cohort (Figure 4E, Supplemental Figure S9F, http://links.lww.com/HC9/B48). Moreover, disease-free survival and progression-free survival in the TCGA cohort also validated this trend. (Supplemental Figures S9G, H, http://links.lww.com/HC9/B48). Furthermore, in the GSE104580 cohort, the estimated proportion of NABP1+ malignant hepatocytes predicted TACE response with an AUC value of 0.661 (95% CI: 0.573–0.749), indicating its relatively accurate predictive performance (Figure 4F).

### Inflammatory NABP1+ malignant hepatocytes showed enhanced interactions with neutrophils following TACE

**FIGURE 5:**
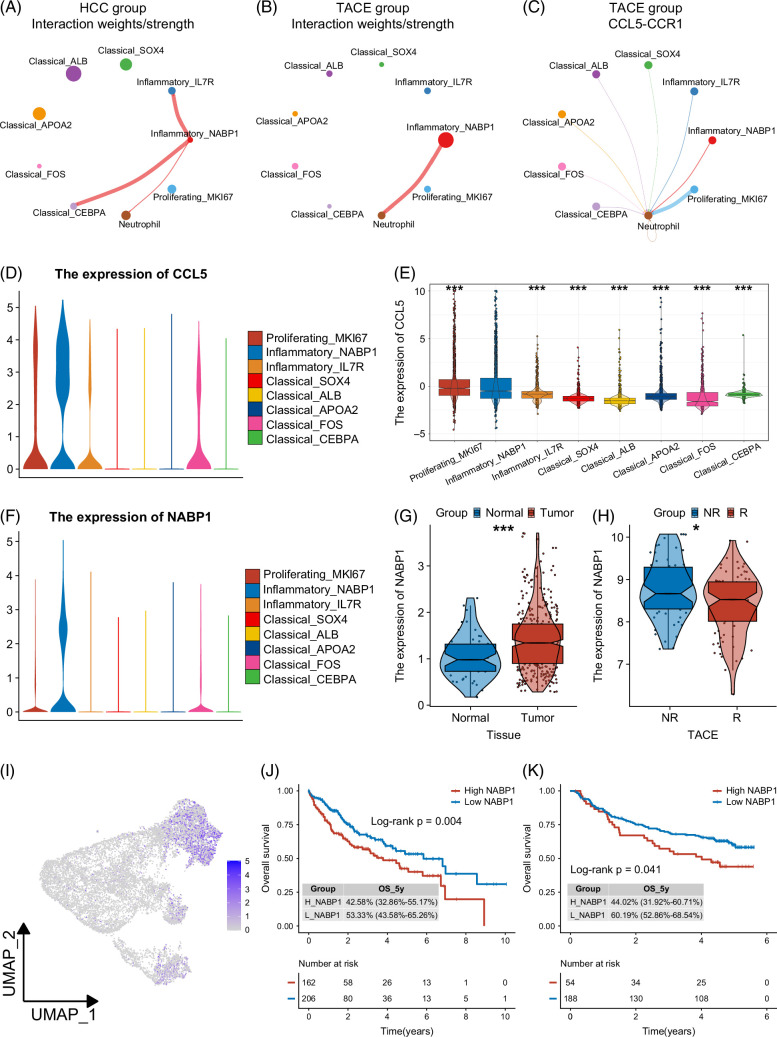
The interactions between NABP1+ malignant hepatocytes and neutrophils and NABP1 is a candidate biomarker. (A, B) Interaction weights of NABP1+ malignant hepatocytes on 8 malignant hepatocyte subsets and neutrophils in the HCC (A) and TACE (B) groups. (C) The interactions of the CCL5-CCR1 ligand-receptor pair between 8 malignant hepatocyte subsets and neutrophils in the TACE group. (D) The expression level of CCL5 in 8 malignant hepatocyte subsets. (E) Boxplot of CCL5 expression levels in 8 malignant hepatocyte subsets. (F) The expression level of NABP1 in 8 malignant hepatocyte subsets. (G) The expression level of NABP1 in tumor and normal samples in the TCGA cohort. (H) The expression level of NABP1 in TACE-responsive and nonresponsive samples in the GSE104580 cohort. (I) The UMAP plot of NABP1 expression in 8 malignant hepatocyte subsets. (J, K) Kaplan-Meier survival curve for OS between the high and low NABP1 expression groups in the TCGA (J) and GSE14520 (K) cohorts. *p < 0.05, **p < 0.01, ***p < 0.001. Abbreviations: OS, overall survival; TACE, transarterial chemoembolization; TCGA, The Cancer Genome Atlas; UMAP, uniform manifold approximation and projection.

Recently, the vital role of neutrophils in triggering inflammation and promoting HCC progression and metastasis has attracted increasing attention; we further performed cell-cell communication analysis between malignant hepatocytes and neutrophils.^33,34^ Interestingly, although the interactions between NABP1+ malignant hepatocytes and IL7R+ malignant hepatocytes, and CEBPA+ malignant hepatocytes were absent, the interactions with neutrophils were significantly enhanced in the TACE group (Figures 5A, B). Specifically, in addition to the ANXA1-FRR1 interaction present in the HCC group, there was a strong interaction between NABP1+ malignant hepatocytes and neutrophils in the TACE group on CCL5-CCR1 signaling (Figure 5C, Supplemental Figures S10A, B, http://links.lww.com/HC9/B48). Moreover, among 8 malignant hepatocyte subsets, NABP1+ malignant hepatocytes possessed a higher CCL5 expression level second only to proliferating cells, and CCR1 expression level on neutrophils was also higher compared with other myeloid cells except monocytes (Figures 5D, E, Supplemental Figure S10C, http://links.lww.com/HC9/B48).

### The signature gene NABP1 is a candidate biomarker for HCC

Next, based on clinical translation, we investigated whether NABP1 could replace this estimated proportion to serve as an eligible biomarker from the following 5 aspects. In the scRNA-seq data, the expression level of NABP1 was highest in NABP1+ malignant hepatocytes (Figures 5F, I, Supplemental Figure S10D, http://links.lww.com/HC9/B48). In addition, the estimated proportion of NABP1+ malignant hepatocytes was significantly positively correlated with NABP1 expression in the TCGA and GSE14520 cohorts (Supplemental Figures S11A, B, http://links.lww.com/HC9/B48). In parallel, NABP1 was significantly overexpressed in the HCC tissues (Figure 5G, Supplemental Figure S11C, http://links.lww.com/HC9/B48). Moreover, high NABP1 expression was distinctly correlated with shorter OS, progression-free survival, and disease-specific survival in the TCGA cohort, and OS and relapse-free survival in the GSE14520 cohort (Figures 5J, K, Supplemental Figures S11D–F, http://links.lww.com/HC9/B48). Most importantly, high NABP1 expression levels corresponded to poorer TACE responsiveness in the GSE104580 cohort (Figure 5H).

In contrast, IL7R and PTPRC, 2 other markers of inflammatory NABP1+ malignant hepatocytes, showed no similar trend. In addition to NABP1+ malignant hepatocytes, IL7R and PTPRC were expressed in IL7R+ malignant hepatocytes, FOS+ malignant hepatocytes, and MKI67+ malignant hepatocytes (Supplemental Figures S12A–F, http://links.lww.com/HC9/B48). Moreover, the results of 2 bulk cohorts, TCGA and GSE14520, showed no significant correlation and weak positive correlation between the expression levels of IL7R and PTPRC, and the estimated proportion of NABP1+ malignant hepatocytes, respectively (Supplemental Figures S12G–J, http://links.lww.com/HC9/B48). In addition, unlike NABP1, the expression of IL7R and PTPRC was significantly lower in tumor tissues (Supplemental Figures S12K–N, http://links.lww.com/HC9/B48). In terms of prognosis, IL7R and PTPRC were overwhelmingly protective factors in HCC, but not all of them were statistically significant (Supplemental Figures S13A–J, http://links.lww.com/HC9/B48). Disappointingly, there was no significant difference in the expression levels of IL7R and PTPRC between the TACE-responsive and nonresponsive groups (Supplemental Figures S13K, L, http://links.lww.com/HC9/B48).

### Biological validation of NABP1 function in HCC

**FIGURE 6:**
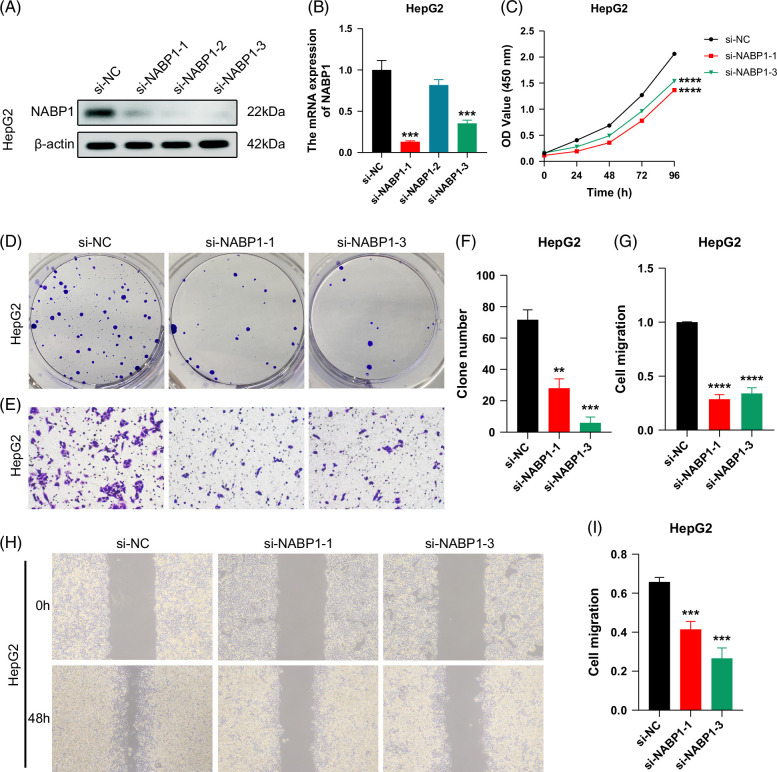
Biological experimental validation of the NABP1 function in HCC. (A) Western blotting results of NABP1 expression in the NC and siRNA groups in the HepG2 cell line. (B) The qRT-PCR results of NABP1 expression in the NC and siRNA groups in the HepG2 cell line. (C) The CCK-8 assay confirmed that the proliferation ability of the HepG2 cell line was significantly inhibited in the si-NABP1 group compared with the NC group. (D) The colony‐forming assay of the HepG2 cell line supported that the sphere-forming capability was worse in the si-NABP1 group than in the NC group. (E) The Transwell assay of the HepG2 cell line supported the migration ability was worse in the si-NABP1 group than in the NC group. (F, G) Clone numbers of colony‐forming assay (F) and relative migration percentage of Transwell assay (G) were compared between the NC and si-NABP1 groups in the HepG2 cell line. (H) The scratch-wound healing assay suggested that the migration ability of HepG2 cells in the si-NABP1 group was inhibited compared with that in the NC group. (I) The relative migration percentage of the scratch-wound healing assay was compared between the NC and si-NABP1 groups in the HepG2 cell line. **p < 0.01, ***p < 0.001, ****p < 0.0001. Abbreviations: CCK-8, cell counting kit-8; NC, negative control; qRT-PCR, quantitative real-time PCR.

In vitro experiments, quantitative real-time PCR and western blotting results consistently demonstrated that our si-NABP1-1 and si-NABP1-3 were successfully transfected (Figures 6A, B). The CCK-8 assay showed that the proliferation ability of HCC cells was significantly decreased after the knockdown of NABP1 (Figure 6C). In the colony‐forming assay, the sphere-forming capability of HCC cells from both si-NABP1 groups was notably inhibited (Figures 6D, F). Furthermore, the results of Transwell and scratch-wound healing assays also unanimously confirmed that the knockdown of NABP1 dramatically diminished the migration ability of HCC cells (Figures 6E, G–I). The results suggest that NABP1 plays a crucial role in the tumorigenesis and progression of HCC and may serve as a promising biomarker and potential therapeutic target.

## DISCUSSION

As TACE is a first-line treatment option for advanced HCC, it is estimated that at least 30% of patients have undergone TACE throughout their disease course.^6^ However, the changed microenvironment caused by TACE remains unclear, and studies on the follow-up treatment strategies for TACE are still insufficient. To bridge this gap, we performed scRNA-seq analysis to delineate the landscape of the altered TME and demonstrated elevated heterogeneity and enhanced proinflammatory responses after TACE. Moreover, we found that the interactions between inflammatory NABP1+ malignant hepatocytes and neutrophils, and neutrophils and CD8+ T cells, may accelerate the shift between CD8_Tem and CD8_Teff. Integrating the bulk cohorts, we further confirmed that the increased NABP1+ malignant hepatocytes were related to poorer TACE response and dismal prognosis in patients with HCC, and its biomarker role could be replaced by NABP1. Overall, our findings depicted a global map of altered TME in HBV-related HCC after TACE and suggested that NABP1 may be a potential target for improving TACE efficacy.

Currently, among the few studies on postoperative changes after TACE, Li et al and Xue et al confirmed that corresponding genomic alterations could already be detected in plasma and tissue samples from HCC 1–3 months after TACE.^8,9^ Recently, Tan et al^20^ performed scRNA-seq on CD45+ immune cells that underwent TACE for 2.5 to 3.3 months, followed by surgical resection, and found that TACE caused a decrease in the CD8+ T cells. Our study focused on the inflammatory period 1–2 months after TACE and demonstrated that TACE induced a relatively activated TME. In particular, the abundance of immune effector cells, such as T cells, NK cells, and B cells increased dramatically. Notably, the interval from TACE to surgery for TACE3, TACE2, and TACE1 patients was 1.07, 1.2, and 1.77 months, respectively, and the results in Supplemental Figure S2D, http://links.lww.com/HC9/B48, show that the T cells decreased with increasing interval, which also partly corroborated the results of Tan and colleagues.

Given the imperative role of inflammation in HBV-related HCC progression,^35^ our findings indicated that the activated TME in the inflammatory period after TACE might be the main culprit for postoperative progression and relapse, which coincided with the noteworthy upregulation of proinflammatory pathways caused by TACE found by Pinato et al.^13^ It also provides a theoretical basis for the gradual decrease in the effective rate and significant increase in the disease progression rate with increasing TACE times.^36^ Moreover, based on the altered TME landscape after TACE, we found that TACE intervention disrupted the balance between tumor cells and the microenvironment, resulting in a significant increase in tumor heterogeneity, especially malignant hepatocytes. This may also contribute to the acceleration of clonal evolution and subsequent treatment resistance.^37^

Recently, several studies have successively certified the crucial role of CCL5 in immune escape, metastasis and resistance of HCC.^38–41^ The interactions between NABP1+ malignant hepatocytes and neutrophils in the TACE group were distinctly increased in the CCL pathways, especially CCL5-CCR1. This finding suggests that TACE may strengthen the crosstalk between tumor cells and neutrophils and then mediate subsequent responses. Furthermore, the important role of the CXCL signaling pathway in tumorigenesis, progression, and metastasis has been widely recognized.^28–30^ Research by Gao et al^31^ also demonstrated that increases in the CXCL16-CXCR6 pathway contributed to proinflammatory TME formation and drove metastasis in HCC. Therefore, the enhanced interactions among NABP1+ malignant hepatocytes, neutrophils, and CD8+ T cells after TACE may be the culprits for postoperative progression and relapse, and potential therapeutic targets in HCC.

In recent years, many studies have reported a dual role of neutrophils in HCC.^33,34,42,43^ Neutrophils can mediate interaction networks of the antitumor response or directly participate in killing tumor cells.^42,43^ Conversely, neutrophils can also act as part of a proinflammatory TME to drive carcinogenesis, progression, and resistance.^33,34^ In this study, we found that the markedly enhanced interactions between neutrophils and CD8_Tem and CD8_Teff cells after TACE may accelerate the shift of CD8_Tem toward CD8_Teff, which, together with the NK T cells, may contribute to the killing of the vast majority of classical malignant hepatocytes. A study by Noh et al^44^ confirmed that the IGF2 signaling pathway plays a crucial role in the therapeutic resistance of glioblastoma. In addition, research by Xue et al^45^ has also demonstrated that neutrophils can contribute to the inhibitory TME of HCC by recruiting macrophages. The interactions between neutrophils and macrophages regarding IGF2-IGF2R, which are present in the HCC group, disappear in the TACE group, potentially alleviating the inhibitory TME to some extent. However, the CD8+ T cells gradually decreased or even became lower with time after TACE. Moreover, the interactions of NABP1+ inflammatory malignant hepatocytes, which became the dominant subset after TACE, with neutrophils were notably increased, and together with the enhanced proinflammatory pathways, they may contribute to the progression and dismal prognosis of HCC after TACE.

As the number of TACE procedures increases, the benefit to patients gradually decreases and even leads to the deterioration of liver function.^36,46^ Thus, it is urgent to identify the population who responds to TACE early. Current studies on TACE have mostly focused on the development of cell-free DNA tools by sequencing a few samples such as plasma samples.^6,8–11^ However, these tools are limited by their low sensitivity, specificity, and even stability and have not been applied in clinical practice. Integrating multicenter cohorts, we confirmed that the estimated proportion of NABP1+ malignant hepatocytes was associated with poorer TACE responsiveness and dismal prognosis. Furthermore, our 5-step pipeline further demonstrated its biomarker role could be replaced by NABP1. At present, the role of NABP1 in tumors has not been reported, and only a few studies have focused on DNA damage and repair.^47,48^ Our study verified that knockdown of NABP1 significantly inhibited the proliferation and migration ability of HCC, hinting that it may be a potential biomarker and therapeutic target for predicting TACE responsiveness.

To the best of our knowledge, this study is the first to systematically delineate the global map of the altered TME after TACE; it is also a useful supplement to the immune landscape proposed by Tan et al.^20^ However, some limitations still need to be considered. First, considering the etiological differences between Eastern and Western populations, our study only included patients with HBV-related HCC, and further studies in NAFLD-related HCC and alcohol-associated HCC are needed to confirm. Second, considering that we included only 6 samples, the sample size was relatively small. The satisfactory stability and reproducibility of the results slightly compensated for this deficiency. In addition, the candidate biomarker NABP1 developed through a 5-step pipeline and validated by in vitro biological experiments will need to be verified in vivo and prospective multicenter cohorts. Furthermore, the proinflammatory role of NABP1+ malignant hepatocytes and their potential interactions with neutrophils require further investigation in the future.

In conclusion, our study delineated the landscape of the altered TME after TACE and demonstrated that the elevated tumor heterogeneity, activated proinflammatory microenvironment, and enhanced interactions within the TME caused by TACE may be the culprits for postoperative progression and relapse, and potential therapeutic targets in HCC. NABP1 may become an attractive tool for the identification of patients with HCC sensitive to receiving first-line TACE therapy in clinical practice.

## Supplementary Material
