# Supplementary material for: Enhanced interactions within microenvironment accelerates dismal prognosis in HBV-related HCC after TACE
Source: Hepatol Commun. 2024 Oct 3;8(10):e0548. doi: 10.1097/HC9.0000000000000548 (PMC11458170; doi:10.1097/HC9.0000000000000548)
Supplement: Supplementary file 1 [file hc9-8-e0548-s001.pdf]

Supplemental material for:

Enhanced interactions within microenvironment accelerates dismal prognosis in HBV-related hepatocellular carcinoma after TACE

Libo Wang[1,2†], Jiahui Cao[1†], Zaoqu Liu[3†], Shitao Wu[1], Yin Liu[1], Ruopeng Liang[1,4,5], Rongtao Zhu[1,4,5], Weijie Wang[1,4,5], Jian Li[1,4,5], and Yuling Sun[1,4,5]*

Supplemental material

- Figure S1. Data quality control and cell annotation.
- Figure S2. Landscape of eight major cell types in the HCC and TACE groups.
- Figure S3. Annotation and functional heterogeneity of T cells in HCC after TACE.
- Figure S4. Landscape and functional heterogeneity of nine heterogeneous T-cell subsets in HCC after TACE.
- Figure S5. Landscape of eight myeloid cell subsets in the HCC and TACE groups.
- Figure S6. Increased cell-cell interactions in HCC following TACE.
- Figure S7. Increased cell-cell interactions in distinct T-cell subset in HCC following TACE.
- Figure S8. Interactions of neutrophils and myeloid subsets and functional enrichment analysis of malignant hepatocytes
- Figure S9. Landscape of eight malignant hepatocyte subsets in the two groups and prognostic value of NABP1+ malignant hepatocytes.
- Figure S10. Interactions between malignant hepatocytes and neutrophils and expression of CCR1 and NABP1 in myeloid and malignant hepatocytes.
- Figure S11. Validation of NABP1 as a candidate biomarker.
- Figure S12. Validation of IL7R and PTPRC as candidate biomarkers.
- Figure S13. Prognosis of IL7R and PTPRC and correlation with TACE response.
- Table S1. Clinical information of the six HCC samples used in this study.
- Table S2. The sequences of three NABP1 siRNA vectors.
- Table S3. The sequences of NABP1 primer used for qRT-PCR.

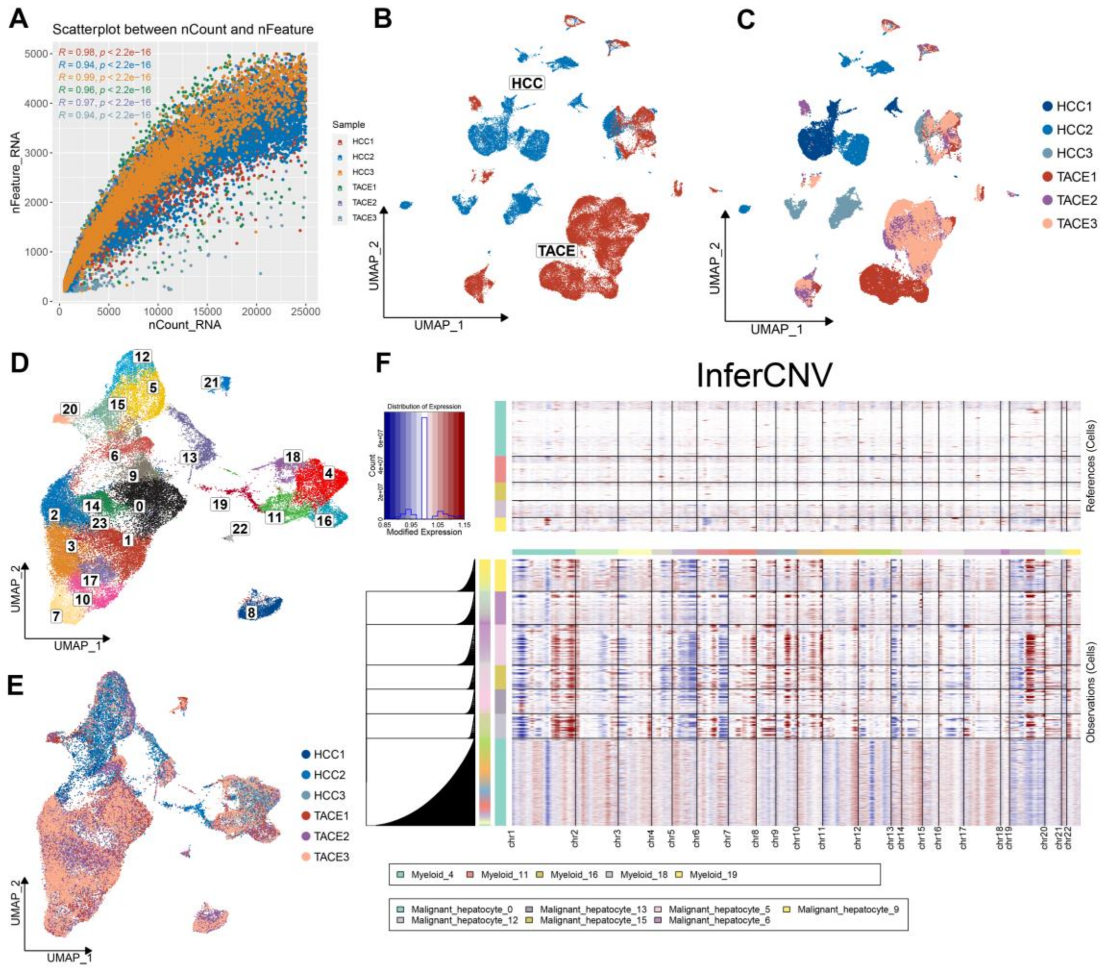

Figure S1. Data quality control and cell annotation. (**A**) The correlation of the total number of UMIs (nCount_RNA) with the detected genes (nFeature_RNA) in six samples. (**B-C**) Uniform manifold approximation and projection (UMAP) plots of eligible cells after quality control in the two groups (**B**) and six samples (**C**). (**D**) The UMAP distribution of 24 clusters that were clustered by integrated cells by CCA and MNNs. (**E**) The UMAP distribution of CCA and MNNs integrated cells in the six samples. (**F**) The distribution of inferred copy number variations (CNVs) on chromosomes for five clusters annotated as malignant hepatocytes compared to four myeloid cell subsets. CCA, canonical correlation analysis; MNNs, mutual nearest neighbors.

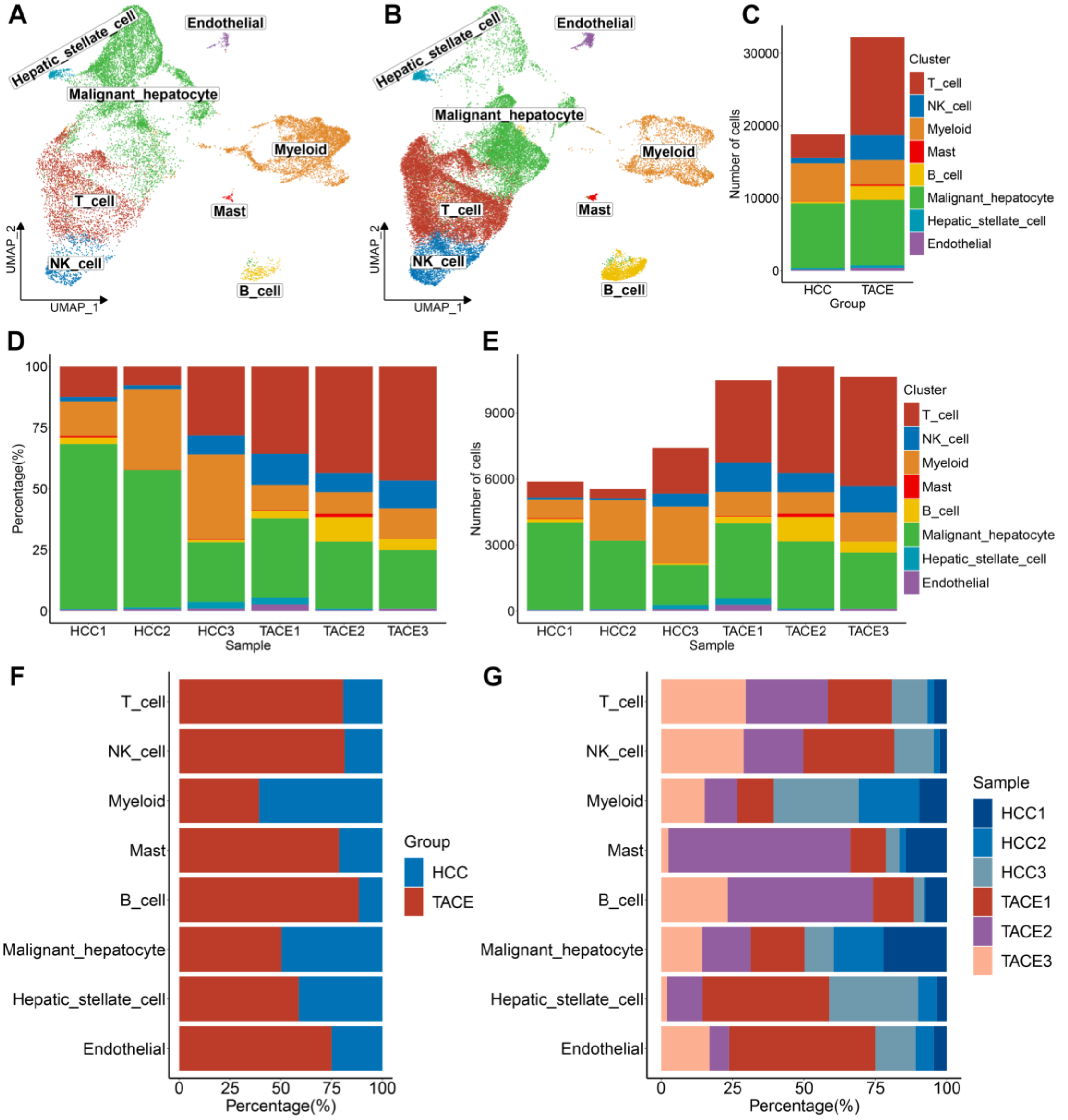

Figure S2. Landscape of eight major cell types in the HCC and TACE groups. (**A-B**) The uniform manifold approximation and projection (UMAP) distribution for eight major cell types in the HCC (**A**) and TACE (**B**) groups. (**C**) The numbers of these eight major cell types in the two groups. (**D**) The percentage of these eight major cell types in the six samples. (**E**) The numbers of these eight major cell types in the six samples. (**F-G**) Barplots of the proportion of these eight major cell types in the two groups (**F**) and six samples (**G**).

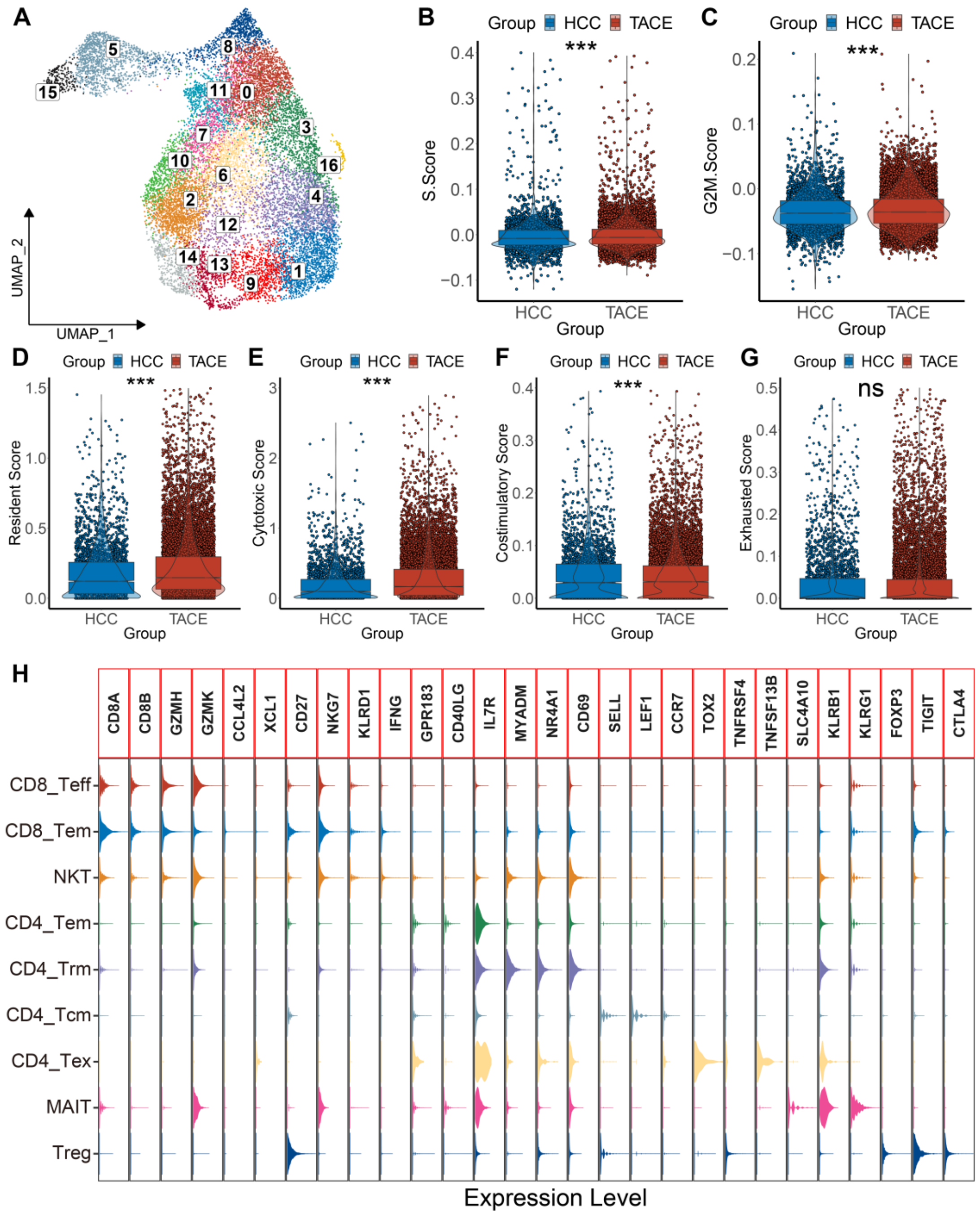

Figure S3. Annotation and functional heterogeneity of T cells in HCC after TACE. (**A**) Uniform manifold approximation and projection (UMAP) plot of 17 clusters clustered by 16,763 T cells. (**B-G**) Comparison of the S.Score (**B**), G2M.Score (**C**), and resident (**D**), cytotoxic (**E**), costimulatory (**F**), and exhausted (**G**) scores of T cells in the HCC and TACE groups. (**H**) The signature markers of these nine heterogeneous T-cell subsets. *$P < 0.05$, **$P < 0.01$, ***$P < 0.001$.

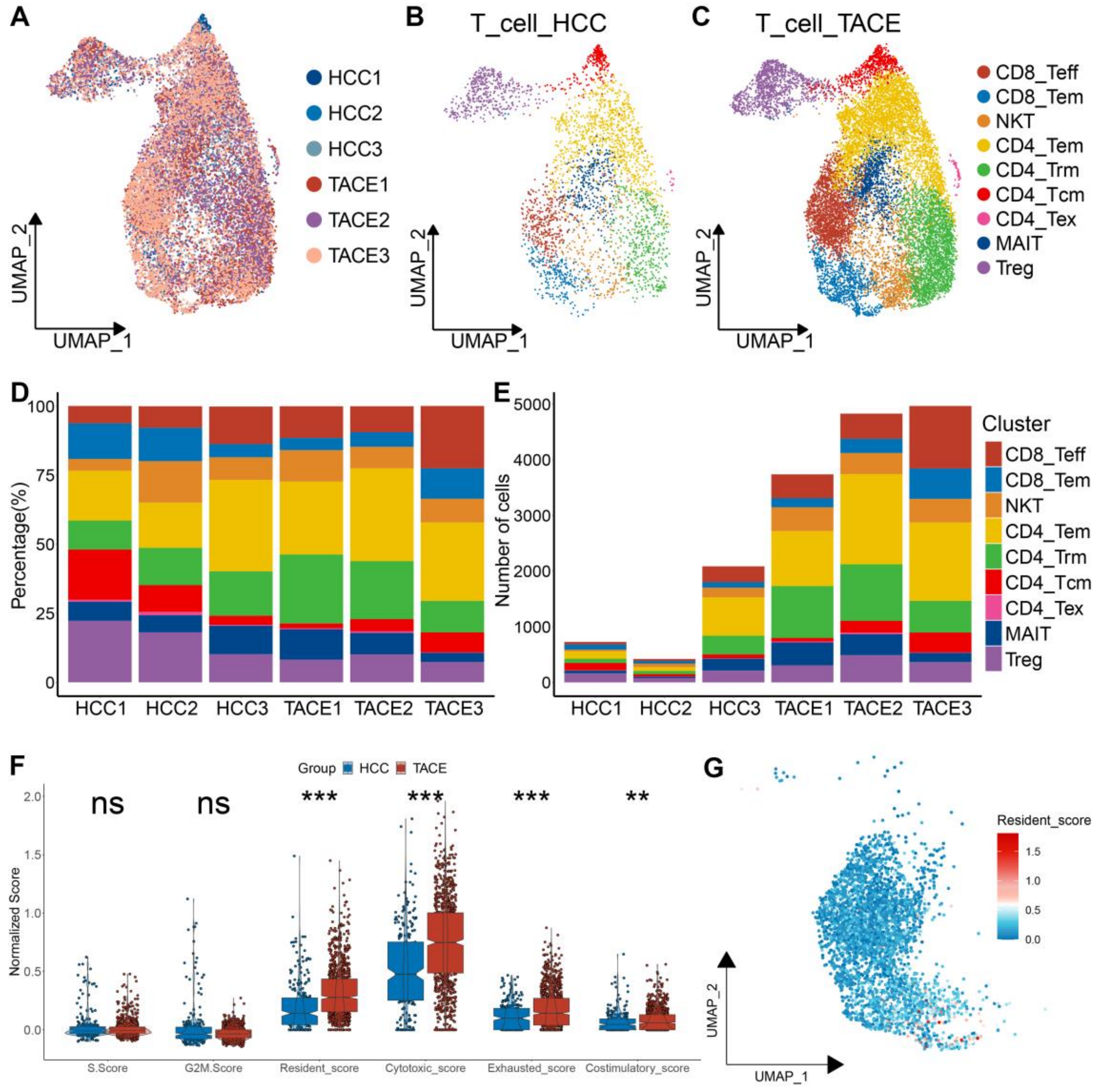

Figure S4. Landscape and functional heterogeneity of nine heterogeneous T-cell subsets in HCC after TACE. (**A**) The uniform manifold approximation and projection (UMAP) distribution of 16,763 T cells in the six samples. (**B-C**) The UMAP distribution for nine T-cell subsets in the HCC (**B**) and TACE (**C**) groups. (**D**) The percentage of these nine T-cell subsets in the six samples. (**E**) The numbers of these nine T-cell subsets in the six samples. (**F**) Comparison of the S.Score, G2M.Score, and resident, cytotoxic, exhausted, and costimulatory scores of CD8+ effector memory T (CD8_Tem) cells in the HCC and TACE groups. (**G**) UMAP plot of resident scores in CD8_Tem and CD8+ effector T (CD8_Teff) cell subsets. *P < 0.05, **P < 0.01, ***P < 0.001.

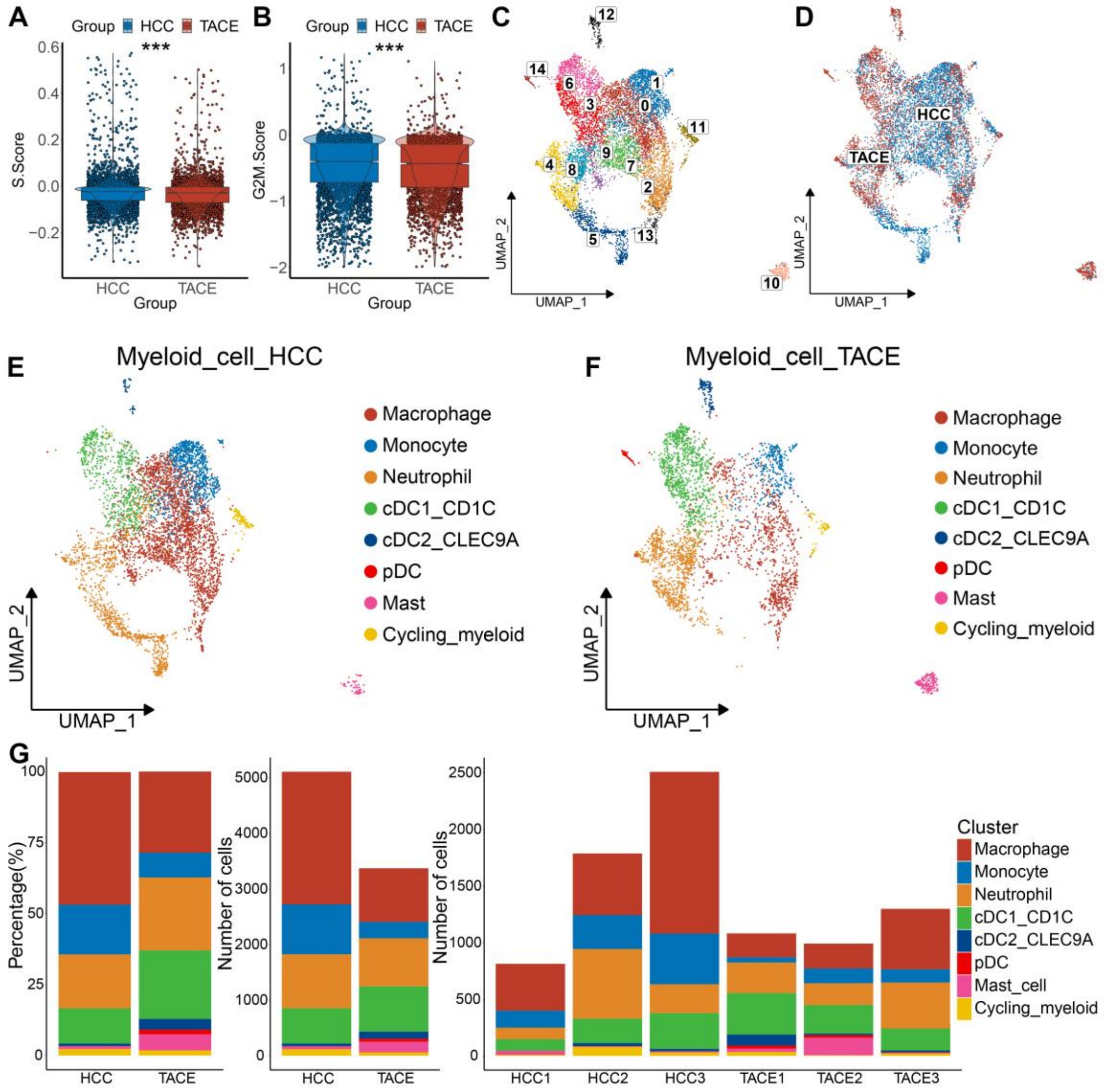

Figure S5. Landscape of eight myeloid cell subsets in the HCC and TACE groups. (**A-B**) Comparison of the S.Score (**A**) and G2M.Score (**B**) of myeloid cells in the HCC and TACE groups. (**C**) Uniform manifold approximation and projection (UMAP) plot of 15 clusters clustered by 8,483 myeloid cells. (**D**) The UMAP distribution of all myeloid cells in the two groups. (**E-F**) The UMAP distribution for eight myeloid cell subsets in the HCC (**E**) and TACE (**F**) groups. (**G**) The percentage and numbers of these eight myeloid cell subsets in the two groups and the six samples. *P < 0.05, **P < 0.01, ***P < 0.001.

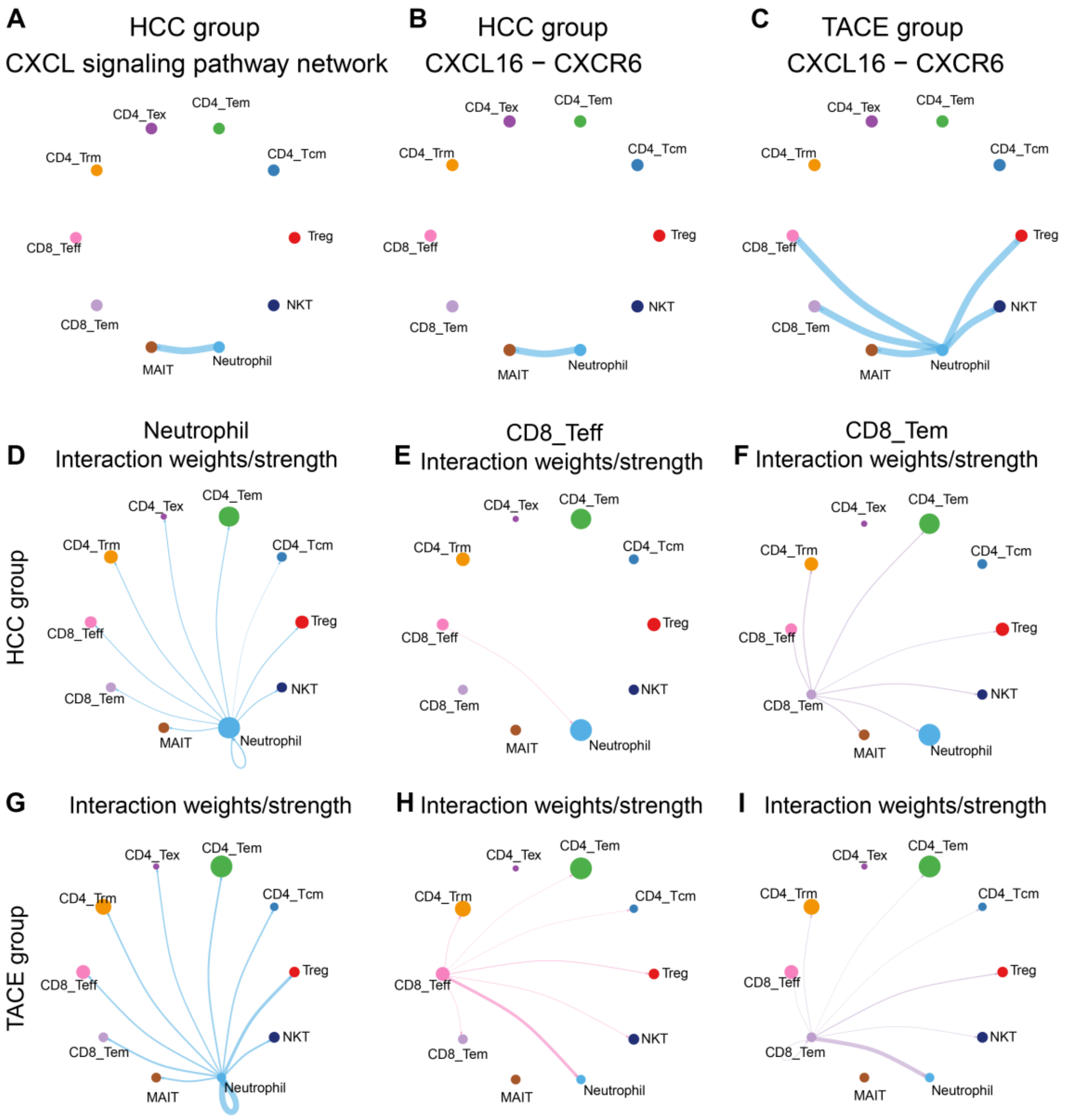

Figure S6. Increased cell-cell interactions in HCC following TACE. (**A**) The CXCL signaling pathway network of neutrophils and nine heterogeneous T-cell subsets in the HCC group. (**B-C**) The interactions of the CXCL16-CXCR6 ligand-receptor pair between neutrophils and nine heterogeneous T-cell subsets in the HCC (**B**) and TACE (**C**) groups. (**D, G**) The interaction weights of neutrophils toward nine heterogeneous T-cell subsets in the HCC (**D**) and TACE (**G**) groups. (**E, H**) The interaction weights of CD8+ effector T (CD8_Teff) cells with the other eight T-cell subsets and neutrophils in the HCC (**E**) and TACE (**H**) groups. (**F, I**) The interaction weights of CD8+ effector memory T (CD8_Tem) cells with the other eight T-cell subsets and neutrophils in the HCC (**F**) and TACE (**I**) groups.

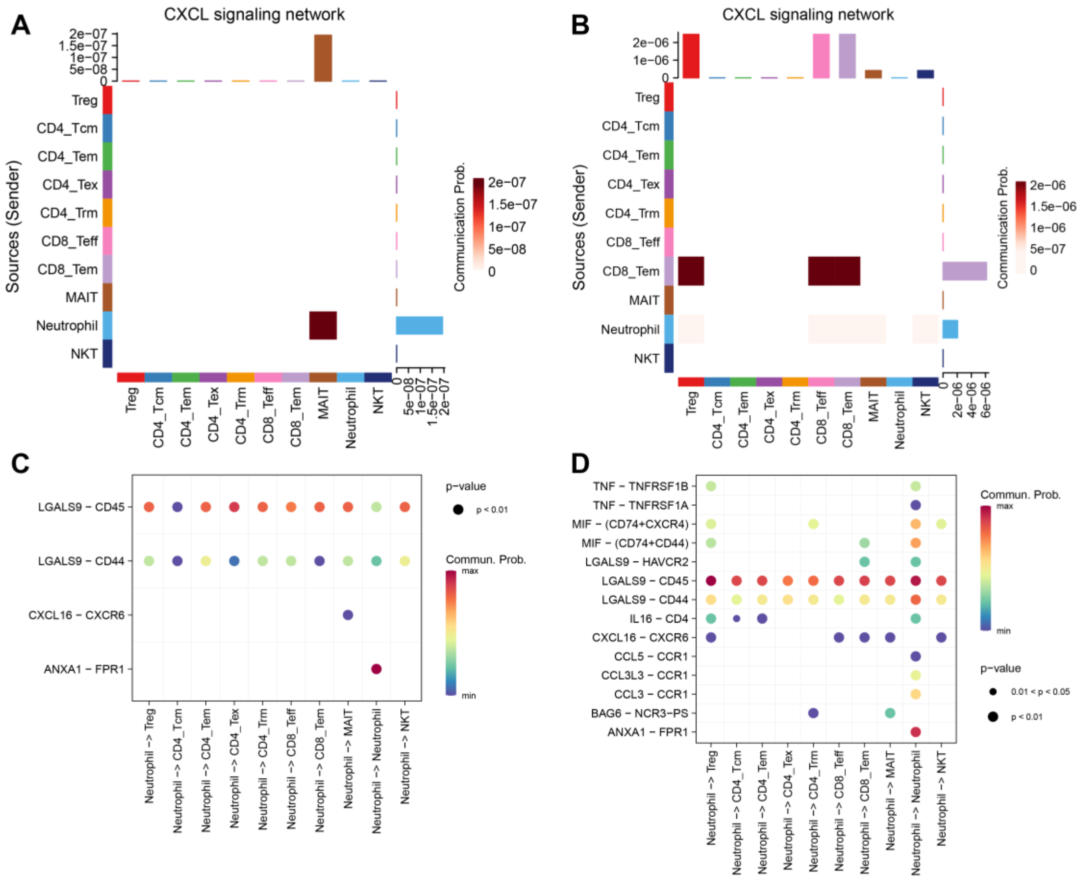

Figure S7. Increased cell-cell interactions in distinct T-cell subset in HCC following TACE. (**A-B**) Overview of sending and receiving signals on the CXCL signaling pathway network between neutrophils and nine heterogeneous T-cell subsets in the HCC (**A**) and TACE (**B**) groups. (**C-D**) Overview of secreted signaling interactions between neutrophils and nine heterogeneous T-cell subsets in the HCC (**C**) and TACE (**D**) groups. P values are indicated by circle size (permutation test).

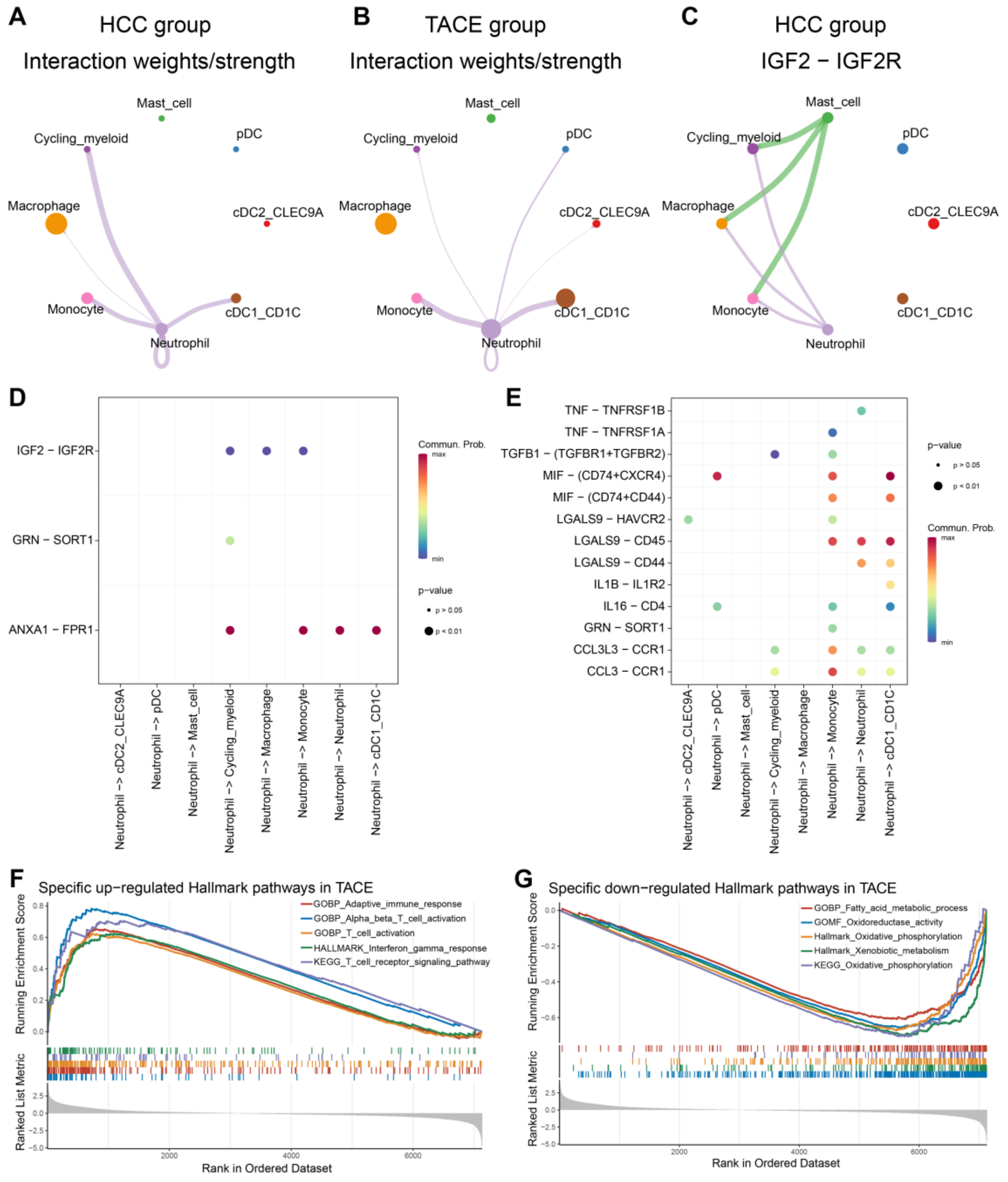

Figure S8. Interactions of neutrophils and myeloid subsets and functional enrichment analysis of malignant hepatocytes. (**A-B**) The interaction weights of neutrophils and myeloid subsets in the HCC (**A**) and TACE (**B**) groups. (**C**) The interactions of the CXCL16-CXCR6 ligand-receptor pair between neutrophils and myeloid subsets in the HCC group. (**D-E**) Overview of secreted signaling interactions between neutrophils and myeloid subsets in the HCC (**D**) and TACE (**E**) groups. P values are indicated by circle size (permutation test). (**F-G**) The top 5 upregulated (**F**) and downregulated (**G**) pathways identified by gene set enrichment analysis (GSEA) in malignant hepatocytes from the TACE group compared to the HCC group.

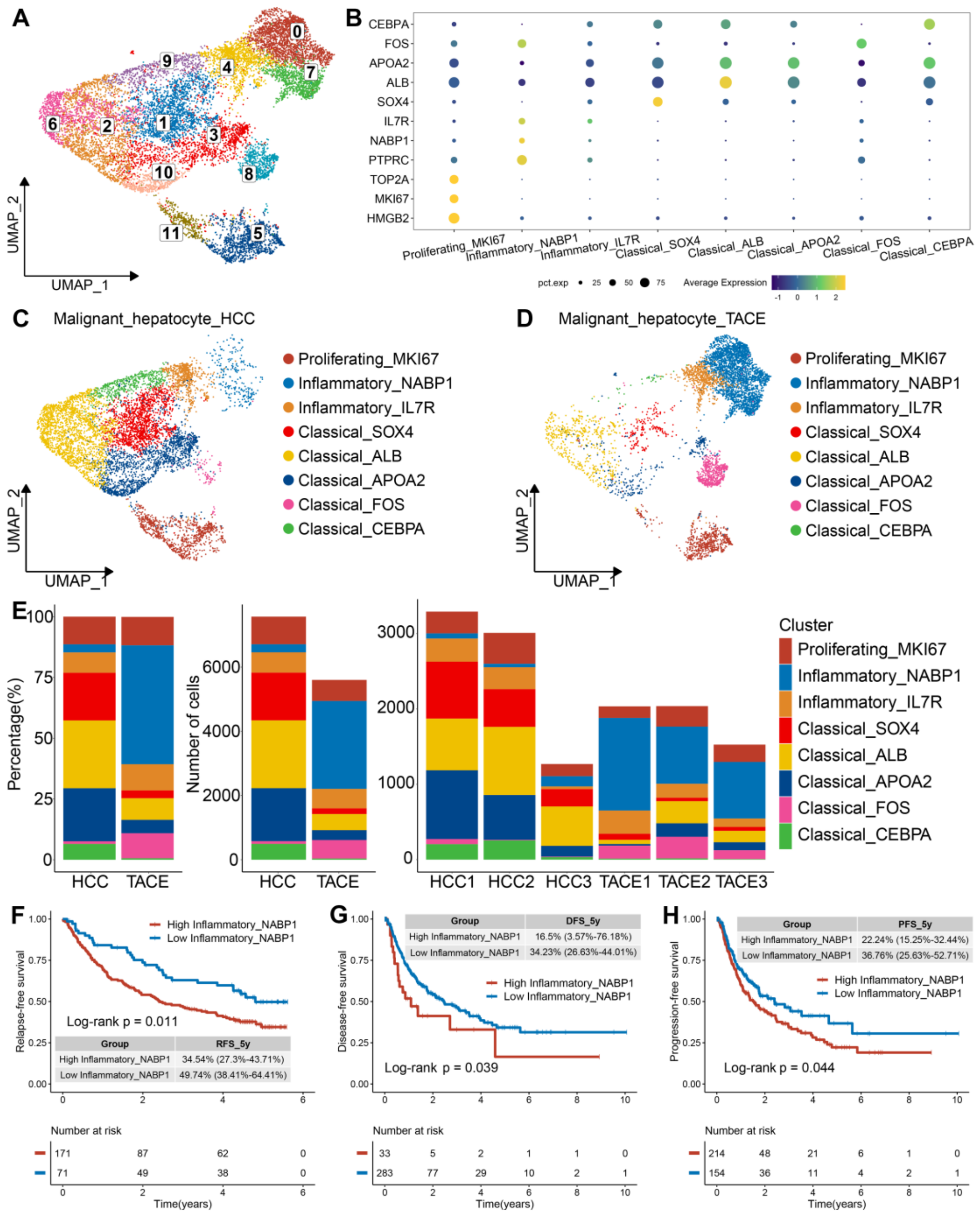

Figure S9. Landscape of eight malignant hepatocyte subsets in the two groups and prognostic value of NABP1+ malignant hepatocytes. (**A**). Uniform manifold approximation and projection (UMAP) plot of eight clusters clustered by 13,172 malignant hepatocytes. (**B**) The signature markers of eight malignant hepatocyte subsets. (**C-D**) The UMAP distribution for eight malignant hepatocyte subsets in the HCC (**C**) and TACE (**D**) groups. (**E**) The percentage and numbers of these eight malignant hepatocyte subsets in the two groups and the six samples. (**F**) Kaplan-Meier survival curve for relapse-free survival (RFS) between groups with high and low estimated proportions of NABP1+ malignant hepatocytes in the GSE14520 cohort. (**G-H**) Kaplan-Meier survival curve for disease-free survival (DFS, **G**) and progression-free survival (PFS, **H**) between groups with high and low estimated proportions of NABP1+ malignant hepatocytes in the TCGA cohort.

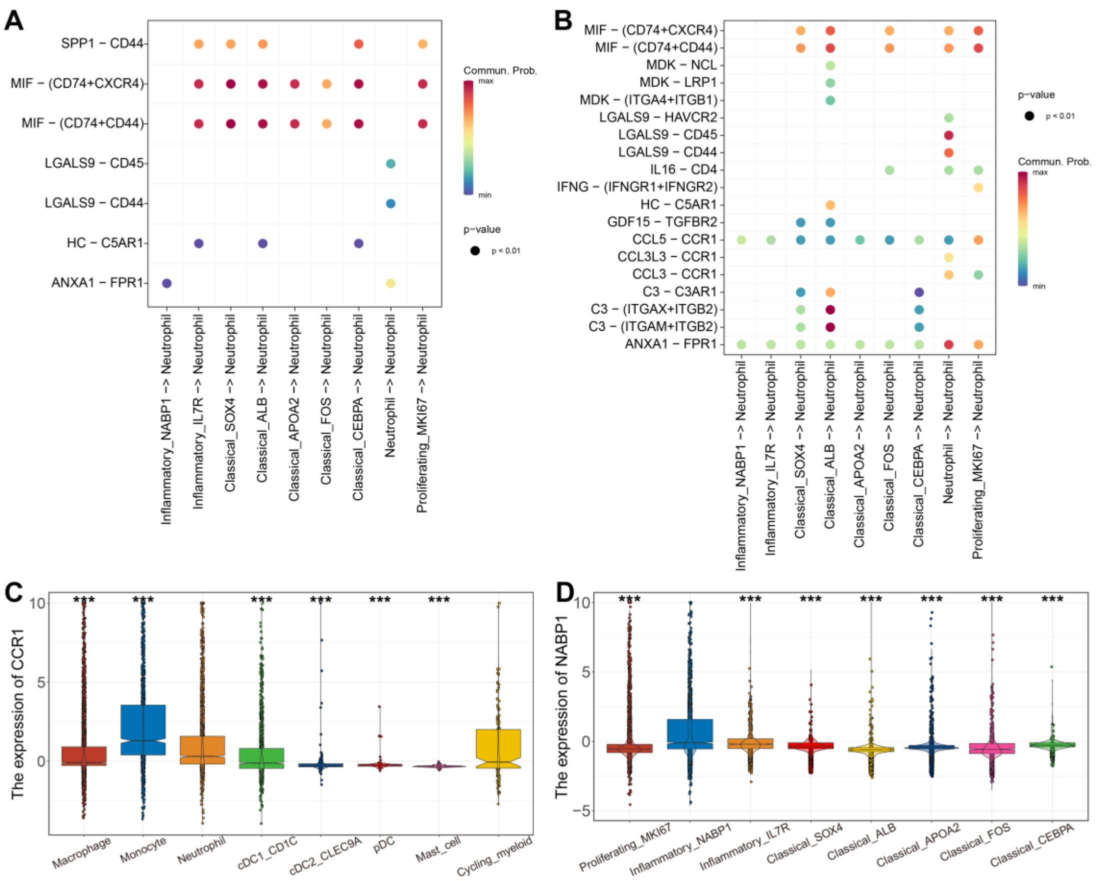

Figure S10. Interactions between malignant hepatocytes and neutrophils and expression of CCR1 and NABP1 in myeloid and malignant hepatocytes. (**A-B**) Overview of secreted signaling interactions between malignant hepatocyte subsets and neutrophils in the HCC (**A**) and TACE (**B**) groups. P values are indicated by circle size (permutation test). (**C**) Boxplot of CCR1 expression levels in eight myeloid subsets. (**D**) Boxplot of NABP1 expression levels in eight malignant hepatocyte subsets. *P < 0.05, **P < 0.01, ***P < 0.001.

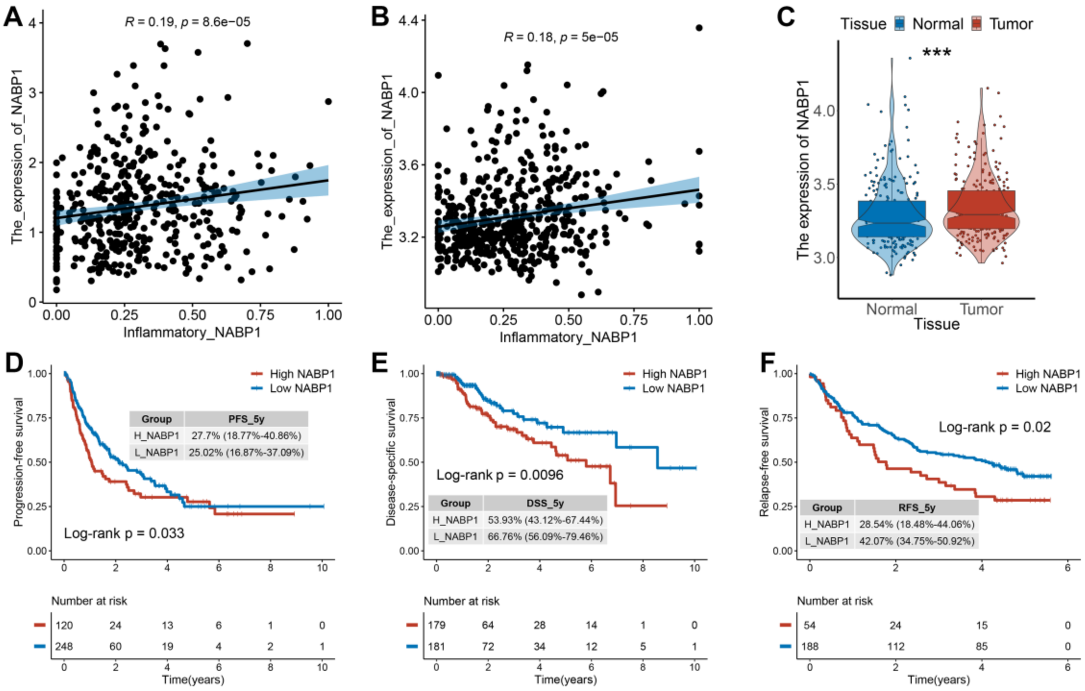

Figure S11. Validation of NABP1 as a candidate biomarker. (**A-B**) The correlation of the estimated proportion of NABP1+ malignant hepatocytes with NABP1 expression in the TCGA (**A**) and GSE14520 (**B**) cohorts. (**C**) The expression level of NABP1 in tumor and normal samples in the GSE14520 cohort. (**D-E**) Kaplan-Meier survival curves for progression-free survival (PFS, **D**) and disease-specific survival (DSS, **E**) between the high and low NABP1 expression groups in the TCGA cohort. (**F**) Kaplan-Meier survival curve for relapse-free survival (RFS) between the high and low NABP1 expression groups in the GSE14520 cohort.

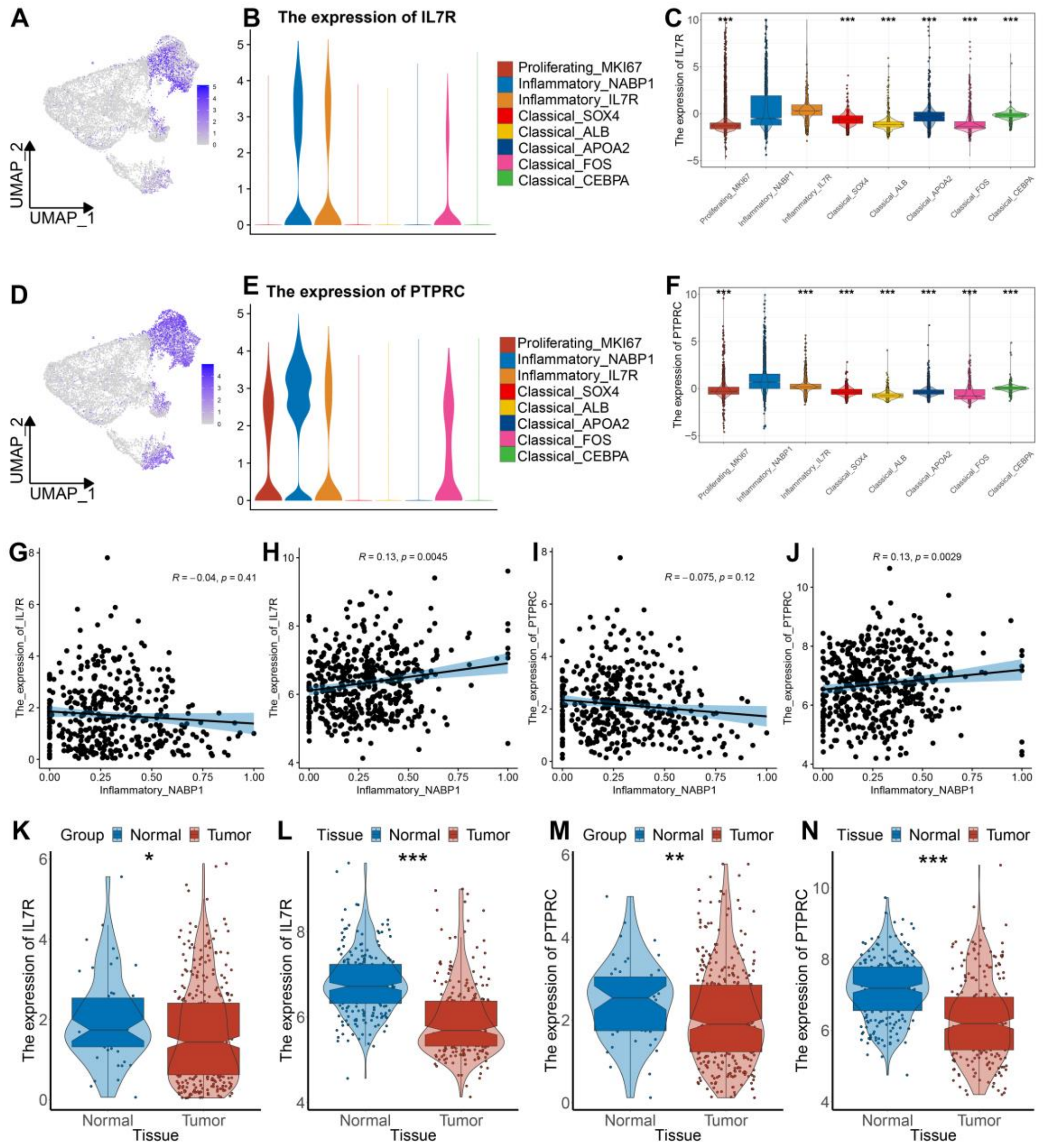

Figure S12. Validation of IL7R and PTPRC as candidate biomarkers. (**A-C**) The uniform manifold approximation and projection (UMAP, **A**), violin (**B**), and boxplot (**C**) of IL7R expression levels in eight malignant hepatocyte subsets. (**D-F**) The uniform manifold approximation and projection (UMAP, **D**), violin (**E**), and boxplot (**F**) of PTPRC expression levels in eight malignant hepatocyte subsets. (**G-H**) The correlation of the estimated proportion of NABP1+ malignant hepatocytes with IL7R expression in the TCGA (**G**) and GSE14520 (**H**) cohorts. (**I-J**) The correlation of the estimated proportion of NABP1+ malignant hepatocytes with PTPRC expression in the TCGA (**I**) and GSE14520 (**J**) cohorts. (**K-L**) The expression level of IL7R in tumor and normal samples in the TCGA (**K**) and GSE14520 (**L**)cohort. (**M-N**) The expression level of PTPRC in tumor and normal samples in the TCGA (**M**) and GSE14520 (**N**)cohort. *P <0.05, **P <0.01, ***P <0.001.

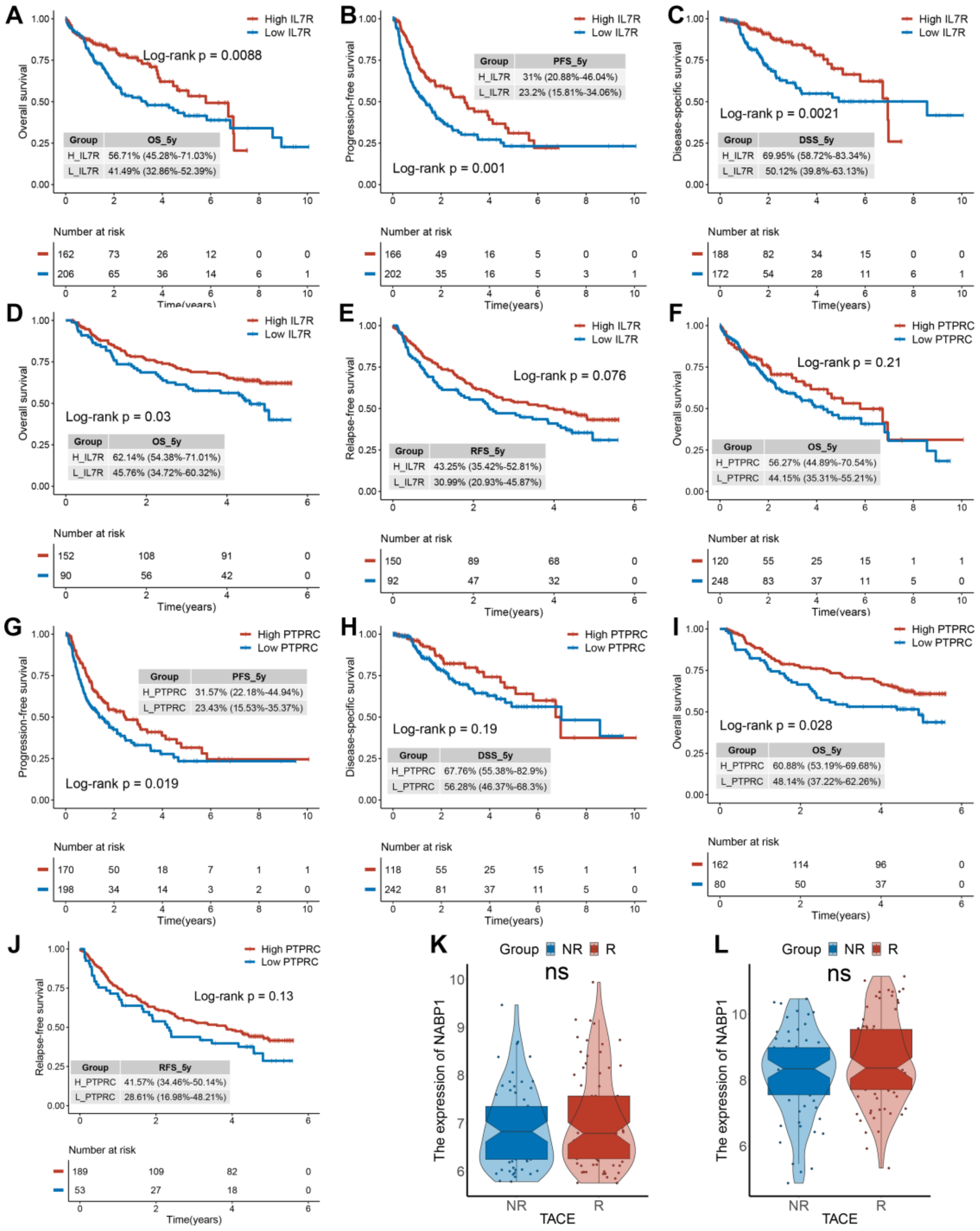

Figure S13. Prognosis of IL7R and PTPRC and correlation with TACE response. (**A-C**) Kaplan-Meier survival curves for overall survival (OS, **A**), progression-free survival (PFS, **B**) and disease-specific survival (DSS, **C**) between the high and low IL7R expression groups in the TCGA cohort. (**D-E**) Kaplan-Meier survival curve for OS (**D**) and relapse-free survival (RFS, **E**) between the high and low IL7R expression groups in the GSE14520 cohort. (**F-H**) Kaplan-Meier survival curves for OS (**F**), PFS (**G**) and DSS (**H**) between the high and low PTPRC expression groups in the TCGA cohort. (**I-J**) Kaplan-Meier survival curve for OS (**I**) and RFS (**J**) between the high and low PTPRC expression groups in the GSE14520 cohort. (**K-L**) The expression level of IL7R (**K**) and PTPRC (**L**) in TACE-responsive and non-responsive samples in the GSE104580 cohort.

Table S1. Clinical information of the six HCC samples used in this study.

Patient _ID	Sex	Age	HBsAg (IU/mL)	Aminoleucine transferase (ALT, U/L)	Aspartate transaminase (AST, U/L)	γ-glutamyl transpeptidase (GGT, U/L)	Total bilirubin (TBIL, μmol/L)	Liver function (Child-Pugh)	Number of foci	Tumor_size_diameter (mm)	Interval from TACE to surgery (months)
TACE1	Male	39	270.895(+)	302	257	25	19.5	A	2	45	1.77
TACE2	Male	51	330.403(+)	21	31	74	111.2	A	1	112	1.2
TACE3	Male	50	247.448(+)	42	40	207	19.2	A	1	180	1.07
HCC1	Male	56	1342(+)	14	18	61	5.9	A	2	66	--
HCC2	Male	46	391.543(+)	32	44	142	11.9	A	2	105	--
HCC3	Male	60	196.637(+)	14	22	36	15.8	A	1	36	--

Table S2. The sequences of three NABP1 siRNA vectors

Vector	Sequence (5' to 3')
si-NABP1-1	GGGTACAGGTACATTTGGA
si-NABP1-2	GTGGAAAGGATGTCTGACA
si-NABP1-3	AGGAACAGCTAGTAATCAA

Table S3. The sequences of NABP1 primer used for qRT-PCR

Gene name	Forward primer	Reverse primer
β-actin	CATGTACGTTGCTATCCAGGC	CTCCTTAATGTCACGCACGAT
NABP1	CAGTGAACCCAACCCAGATTATC	GGTCCAAATGTACCTGTACCCAT
